# Usability of Telehealth Systems for Noncommunicable Diseases in Primary Care From the COVID-19 Pandemic Onward: Systematic Review

**DOI:** 10.2196/44209

**Published:** 2023-03-16

**Authors:** Roberta Lins Gonçalves, Adriana Silvina Pagano, Zilma Silveira Nogueira Reis, Ken Brackstone, Tainá Costa Pereira Lopes, Sarah Almeida Cordeiro, Julia Macedo Nunes, Seth Kwaku Afagbedzi, Michael Head, Wagner Meira Jr, James Batchelor, Antônio Luiz Pinho Ribeiro

**Affiliations:** 1 Hospital das Clínicas da Universidade Federal de Minas Gerais Universidade Federal de Minas Gerais Belo Horizonte Brazil; 2 Universidade Federal do Amazonas Faculdade de Educação Física e Fisioterapia Manaus Brazil; 3 Department of Linguistics Universidade Federal de Minas Gerais Belo Horizonte Brazil; 4 University of Southampton Southampton United Kingdom; 5 University of Ghana Legon-Accra Ghana

**Keywords:** health care professional, telehealth, noncommunicable disease, usability, COVID-19 pandemic, COVID-19

## Abstract

**Background:**

During the COVID-19 pandemic, telehealth was expanded without the opportunity to extensively evaluate the adopted technology’s usability.

**Objective:**

We aimed to synthesize evidence on health professionals’ perceptions regarding the usability of telehealth systems in the primary care of individuals with noncommunicable diseases (NCDs; hypertension and diabetes) from the COVID-19 pandemic onward.

**Methods:**

A systematic review was performed of clinical trials, prospective cohort studies, retrospective observational studies, and studies that used qualitative data collection and analysis methods published in English, Spanish, and Portuguese from March 2020 onward. The databases queried were MEDLINE, Embase, BIREME, IEEE Xplore, BVS, Google Scholar, and grey literature. Studies involving health professionals who used telehealth systems in primary care and managed patients with NCDs from the COVID-19 pandemic onward were considered eligible. Titles, abstracts, and full texts were reviewed. Data were extracted to provide a narrative qualitative evidence synthesis of the included articles. The risk of bias and methodological quality of the included studies were analyzed. The primary outcome was the usability of telehealth systems, while the secondary outcomes were satisfaction and the contexts in which the telehealth system was used.

**Results:**

We included 11 of 417 retrieved studies, which had data from 248 health care professionals. These health care professionals were mostly doctors and nurses with prior experience in telehealth in high- and middle-income countries. Overall, 9 studies (82%) were qualitative studies and 2 (18%) were quasiexperimental or multisite trial studies. Moreover, 7 studies (64%) addressed diabetes, 1 (9%) addressed diabetes and hypertension, and 3 (27%) addressed chronic diseases. Most studies used a survey to assess usability. With a moderate confidence level, we concluded that health professionals considered the usability of telehealth systems to be good and felt comfortable and satisfied. Patients felt satisfied using telehealth. The most important predictor for using digital health technologies was ease of use. The main barriers were technological challenges, connectivity issues, low computer literacy, inability to perform complete physical examination, and lack of training. Although the usability of telehealth systems was considered good, there is a need for research that investigates factors that may influence the perceptions of telehealth usability, such as differences between private and public services; differences in the level of experience of professionals, including professional experience and experience with digital tools; and differences in gender, age groups, occupations, and settings.

**Conclusions:**

The COVID-19 pandemic has generated incredible demand for virtual care. Professionals’ favorable perceptions of the usability of telehealth indicate that it can facilitate access to quality care. Although there are still challenges to telehealth, more than infrastructure challenges, the most reported challenges were related to empowering people for digital health.

**Trial Registration:**

PROSPERO International Prospective Register of Systematic Reviews CRD42021296887; https://www.crd.york.ac.uk/PROSPERO/display_record.php?RecordID=296887

**International Registered Report Identifier (IRRID):**

RR2-10.21801/ppcrj.2022.82.6

## Introduction

Noncommunicable diseases (NCDs) are responsible for at least 71% of global deaths, with hypertension and diabetes being the main diseases [1-3]. Most of these deaths, however, can be prevented with proper treatment [1-3]. These diseases are chronic and progressive, and often require complex and continuous management of physiological, environmental, and behavioral factors [1-4]. Treatment expenses are high for both patients and health services [1-3]. As a result of the COVID-19 pandemic, there was a loss in the follow-up of patients with NCDs due to the increased risk of illness and death from COVID-19 posed by in-person consultation [3-6]. Patients with chronic diseases, such as hypertension and diabetes, are strongly affected by COVID-19. This can be attested by the high prevalence of patients with NCDs and COVID-19 [7]. More consequential, patients with hypertension and diabetes are much more likely to develop a more serious illness or die once infected by the virus when compared to patients who do not have a chronic disease [7].

NCDs are a major challenge for sustainable development. The United Nations (UN) 2030 Agenda for Sustainable Development has committed to developing responses to reduce premature mortality from NCDs by one-third. Innovative and cost-effective solutions that improve the care of people with NCDs have become increasingly relevant [8,9], with telehealth emerging as one of the great alternatives to maintain and enhance health care. Solutions have been implemented even in diverse geographical areas, thus enabling clinical and nonclinical, and synchronous and asynchronous services of different modalities and different functions [10]. Telehealth is a revolutionary patient management approach combining various forms of information communication technologies [8,10,11]. It allows access to health assessment, diagnosis, intervention, consultation, rehabilitation, supervision, education, and information at a distance [8,11]. Primary care practices, especially for NCDs, quickly resorted to telehealth to deal with the COVID-19 pandemic [12]. However, it is unclear whether telehealth will continue to be widely used after the pandemic.

Although numerous studies have shown that patients are satisfied with telehealth and value its convenience [12-17], data on the assessment of telehealth usability by health professionals who use telehealth systems are scarce [18]. Usability is one of the essential quality attributes of any system and is related to its acceptability by its end users [19-23]. Measuring usability allows for the assessment of user and provider satisfaction and establishes strengths and weaknesses that can help improve the effectiveness of the technology and services provided [19-23]. This information is essential for understanding barriers and strengths that can consolidate telehealth.

Based on this, the main interest of this review was to understand how health care professionals assess a telehealth usability system to provide and support health care services for patients with hypertension and diabetes starting from the COVID-19 pandemic. This study was based on a comprehensive review of scientific publications on the topic.

## Methods

### Study Design

We conducted a systematic review of qualitative research following the methodological recommendations of the Cochrane Collaboration Handbook [24] and reported the findings according to the PRISMA (Preferred Reporting Items for Systematic Reviews and Meta-Analysis) statement [25]. The study protocol was registered with PROSPERO (CRD42021296887) in December 2021 [26] and has been published [27].

### Identification and Selection of Studies

Three independent reviewers performed the literature search using MeSH (Medical Subject Headings) and appropriate keywords for each database. The strategy was initially designed for MEDLINE (via PubMed) and then adapted for use in the following databases: Embase, BIREME, IEEE Xplore, BVS, Google Scholar, and gray literature. The search started in December 2021 and ended at the end of March 2022. Clinical trials, prospective cohort studies, retrospective observational studies, and studies that used qualitative data collection and analysis methods in English, Portuguese, and Spanish were searched. The following filters were used: (1) publication date: 2020, 2021, or within 1 year; (2) language: English, Portuguese, or Spanish; and (3) study type: human studies only. Detailed search strategies are presented in Multimedia Appendix 1.

Eligibility criteria based on the PEOTS (patient/population-exposure-outcome-time-study design) framework and inclusion criteria are shown in Textbox 1. Studies that did not answer our research questions included studies that did not report on telehealth usability by health professionals and incomplete articles. Abstracts, review articles, editorials, blogs, books, academic works, dissertations, theses, duplicate articles, and scientific events were excluded.

**Study inclusion criteria.**

**Inclusion criteria**

1. Participants: All categories of health professionals who work in the care of patients with hypertension and diabetes and used telehealth.
2. Exposure: Telehealth.
3. Primary outcome: Usability or ease of use regarding patient care.
4. Secondary outcome: Satisfaction (acceptance), impression of the patient’s satisfaction, and contexts in which digital tools are used.
5. Time: From March 2020 (from the COVID-19 pandemic onwards).
6. Study design: Clinical trials, prospective cohort studies, retrospective observational studies, and cross-sectional observational studies.

Article search and selection were carried out blindly and independently by 3 evaluators (2 had a first degree in health sciences and 1 had a first degree in applied linguistics) following a 3-phase process that included identification, screening, and inclusion. In the identification phase, a search was carried out in the databases through descriptors and filters. Titles and abstracts of identified studies were searched. Articles deemed potentially relevant from their titles and abstracts were retrieved as full-text articles and evaluated in terms of meeting eligibility requirements. In the screening phase, studies were selected based on their titles, abstracts, and full text according to the inclusion and exclusion criteria. Disagreements among evaluators were discussed until a consensus was reached. In the inclusion phase, the final selection of included studies was performed for qualitative analysis.

To manage the data, including deduplication, the software Rayyan from Qatar Computing Research Institute (QCRI) was used [28]. After the final selection, data were extracted and information about the included studies was organized into a predefined model, which included title, scientific journal, year of publication, study type, sample population, country, study outcomes, and additional findings of interest for the review. Excluded articles were reported, together with the reasons for their exclusion.

### Data Analysis

A narrative qualitative evidence synthesis of the findings from the included articles was performed. Screening, selection, data extraction, and bias assessment of the studies were performed by 2 evaluators (a health professional and a linguist) independently, and the results were compared.

### Characteristics of the Included Studies

A predefined template was used to collect the main characteristics and outcomes of the studies included. A summary of the qualitative findings table of the review has been displayed for an evidence profile [29].

### Risk of Bias and Methodological Quality Analysis

The quality of the selected studies and their results were analyzed. The data for the critical quality analysis were consolidated in the Joanna Briggs Institute (JBI) [30] form for observational studies to assess the methodological quality of the studies and determine the extent of possibilities for bias, conduct, and analysis. The JBI tool comprises the following 8 questions:

1. Were the criteria for inclusion in the sample clearly defined?
2. Were the study subjects and the setting described in detail?
3. Was the exposure measured in a valid and reliable way?
4. Were objective standard criteria used for measurement of the condition?
5. Were the confounding factors identified?
6. Were strategies to deal with confounding factors stated?
7. Were the outcomes measured in a valid and reliable way?
8. Was appropriate statistical analysis used?

The studies were categorized according to the percentage of positive responses to the questions present in the assessment instrument. The risk of bias was considered high when the study had below 49% of responses classified as “yes,” moderate when the study had 50% to 69% of responses classified as “yes,” and low when the study had more than 70% of responses classified as “yes.”

Studies were not excluded based on the assessment of the risk of bias and methodological quality, but we used this information to assess confidence in the synthesis findings as part of the GRADE-CERQual approach (Grading of Recommendations Assessment, Development and Evaluation - Confidence in Evidence from Qualitative Research Reviews) [29].

### Report and Recommendations

The Qualitative Research Checklist of the Critical Appraisal Skills Program (CASP), part of the Oxford Center for Triple Value Healthcare Ltd [31], was used to evaluate the studies qualitatively and systematically by 2 reviewers (a health professional and a linguist) independently (Multimedia Appendix 2). This tool analyzes whether the results of the review are valid, what the results are, and whether the results will be useful locally.

The CASP tool comprises the following 10 questions (answer options: *yes*, *can’t tell*, and *no*):

1. Was there a clear statement of the aims of the research?
2. Is a qualitative methodology appropriate?
3. Was the research design appropriate to address the aims of the research?
4. Was the recruitment strategy appropriate to the aims of the research?
5. Was the data collected in a way that addressed the research issue?
6. Has the relationship between researchers and participants been adequately considered?
7. Have ethical issues been taken into consideration?
8. Was the data analysis sufficiently rigorous?
9. Is there a clear statement of the findings?
10. How valuable is the research?

Two evaluators (a health professional and a linguist) independently analyzed the quality of evidence and strength of recommendation by the GRADE-CERQual approach [32]. This tool is based on a systematic system and transparent framework to assess confidence in individual review results based on consideration of the following 4 components: (1) methodological limitations, (2) coherence, (3) adequacy of data, and (4) relevance.

Methodological limitations refer to whether there is concern with the design or conduct of primary studies that contribute evidence to an individual review finding [33]. Coherence is defined as assessing how clear and cogent the fit is between the data from the primary studies and a review finding that synthesizes that data. By “cogent,” we mean well supported or compelling [34]. Adequacy of data means an overall determination of the degree of richness and quantity of data supporting a review finding [35]. Relevance refers to the extent to which the body of evidence from the primary studies supporting a review finding is applicable to the context (perspective or population, phenomenon of interest, and setting) specified in the review question [36].

The methodological analysis of the included studies was carried out in steps [29]. Step 1 aimed to collect and consider the information necessary to report methodological limitations. Step 2 aimed to assess the body of data that contributed to each review finding and decide whether there was a consensus on methodological limitations. Step 3 aimed to pass judgment on the seriousness of the concerns and justify that.

The result of the recommendation is considered as follows [29]:

- High confidence: if it is highly likely that the outcome of the review is a reasonable representation of the phenomenon of interest. No or very minor concerns regarding methodological limitations/coherence/adequacy/relevance that are unlikely to reduce confidence in the review finding.
- Moderate confidence: if it is likely that the review finding is a reasonable representation of the phenomenon of interest. Minor concerns regarding methodological limitations/coherence/adequacy/relevance that may reduce confidence in the review finding.
- Low confidence: if it is possible that the review finding is a reasonable representation of the phenomenon of interest. Moderate concerns regarding methodological limitations/coherence/adequacy/relevance that will probably reduce confidence in the review finding.
- Very low confidence: if it is not clear whether the review finding is a reasonable representation of the phenomenon of interest. Serious concerns regarding methodological limitations/coherence/adequacy/relevance that are very likely to reduce confidence in the review finding.

## Results

### Included Studies

**Figure 1:**
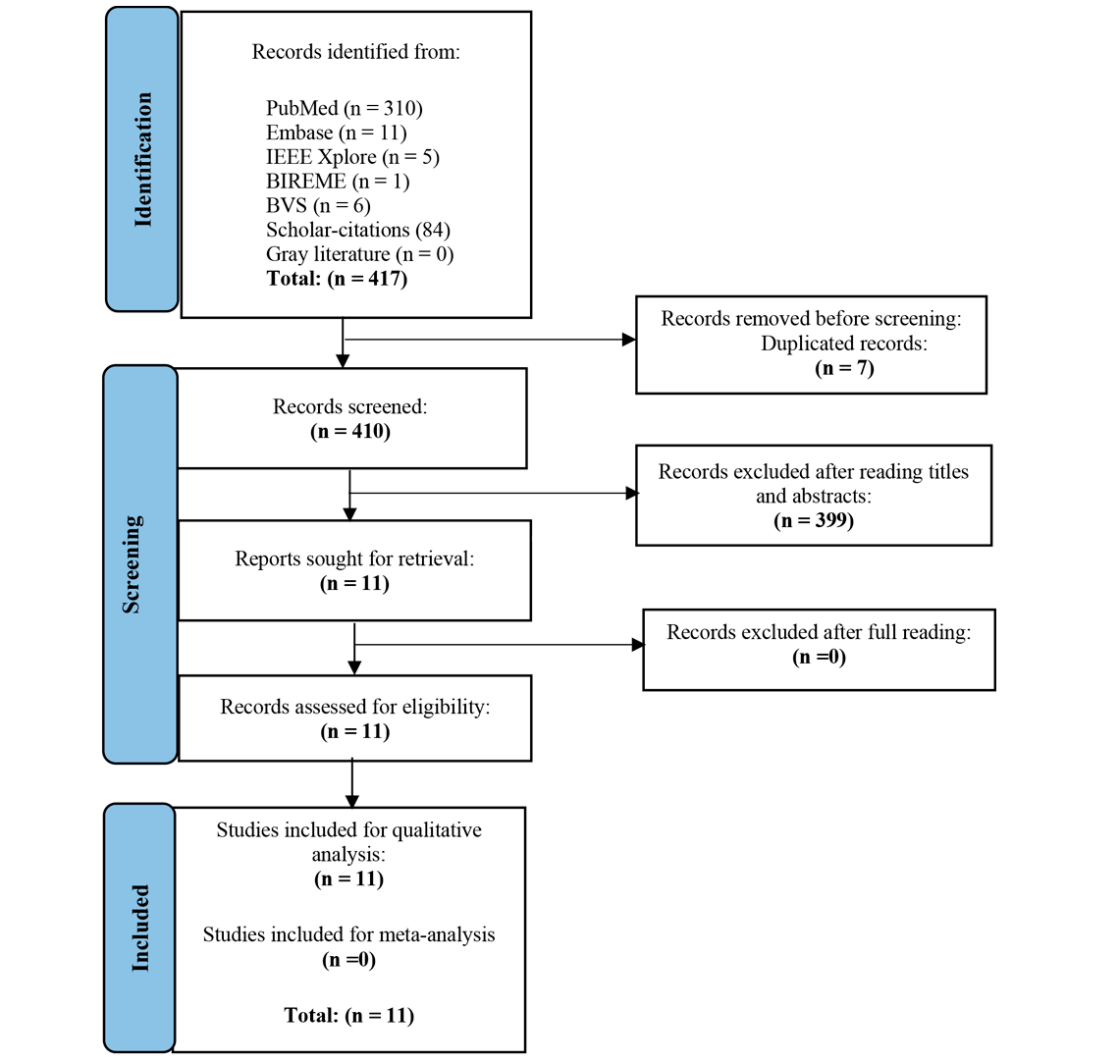
PRISMA flowchart diagram of article selection.

A total of 417 abstracts were selected, and 11 full-text articles were evaluated. Eleven studies met our inclusion criteria. Figure 1 shows our search strategy.

### Excluded Studies

We excluded 399 studies (Multimedia Appendix 3). The reasons for exclusion were different populations than intended, different outcome variables, nonattention to the PEOTS questions (Textbox 1), different study periods, different types of publications, and different languages than stipulated.

### Characteristics of the Included Studies

Table 1 summarizes the results of the individual studies.

**Table 1:** Characteristics of the included studies.

Article title	Population of interest for the review	Outcomes and main findings of interest for the review	Barriers, limitations, and gaps
Views of patients and providers on the use of telemedicine for chronic disease specialty care in the Alaska native population [37]	10 physicians from specialist clinics at Alaska Native Medical Center who used telemedicine to provide care (40% had a lot of experience in telehealth, 30% some, and 30% little). Half of the sample was female and half male. Moreover, 80% were aged 40-59 years and 20% were over 60 years.	Use of video-telemedicine for specialist chronic disease care in the Alaska Tribal Health System, including perceived benefits and barriers, and best uses. The evaluation was carried out through an interview. 40% of health professionals interviewed feel comfortable with video telemedicine, 30% very comfortable, 10% neutral, 20% somewhat uncomfortable, and 0% very uncomfortable.	The barriers identified in telehealth were technical difficulties, the need for prior training of professionals, language barriers, cultural barriers, and technological barriers, especially in elderly patients or patients from remote areas. Other issues were lack of physical contact or the inability to do a hands-on physical examination, scheduling issues, and privacy concerns. The study describes times when telehealth is not seen as beneficial, when the patient needs diagnoses/procedures/medicines that can only be provided in large hospitals, when there is an insurmountable language barrier or cultural barrier, when there are hearing difficulties, when there are new patients/unstable patients/undiagnosed patients, and when it is necessary to discuss sensitive topics.
Criteria for assessing and recommending digital diabetes tools: A Delphi study [38]	15 health care professionals (3 physicians, 10 nurses, 1 nutritionist, and 1 occupational therapist) with diabetes care experience, an average of 19 years of experience in diabetes care working in the 4 health regions of Norway, and an average of 6.8 years of experience with digital tools.	Usability, information quality, data accessibility, adaptability, visual presentation, remote monitoring, and automated recording of data from apps, websites, and visual media (diabetes telehealth) in Norway were assessed by interview (Delphi study). Usability was rated great for apps, websites, and social media.	The authors cite security and privacy as possible barriers to using digital tools.
MoTHer, an mHealth system to support women with gestational diabetes mellitus: Feasibility and acceptability study [39]	Physicians (number not reported)	Satisfaction and ease of use of the mHealth platform (smartphone and internet-based interactive support system) for telemedicine among pregnant women with diabetes mellitus in Australia were assessed using survey questionnaires. Physicians indicated satisfaction and ease of use of the mHealth platform, and feasibly integrated into existing clinical practice.	Health professionals point out technological and connection problems as barriers. The authors also argue that funding models have traditionally not considered the use of telehealth interventions, with the lack of codes and manipulation of reimbursement as barriers to the acceptance of telehealth in public and private health settings. They add that interoperability with existing prevailing health care practices and information technology systems (including data privacy regulations) should be prioritized, emphasizing that technology advances much faster than regulatory reform.
Implementation of digital monitoring services during the COVID-19 pandemic for patients with chronic diseases: Design science approach [40]	53 health care professionals (37 physicians and 16 nurses)	The usefulness of the digital platform (web-based app for teleconsultations, monitoring of patient data, alerts, and therapeutic management) for chronic diseases in Portugal was assessed through an interview. Several physicians and nurses pointed out the user-friendliness of the digital platform.	The authors argue that there is a lack of guidelines on remote patient management using digital health solutions, adequate technological literacy, and internet access. They cite the possibility of misdiagnosis, equipment malfunction, and privacy violations as potential risks. They address the need for adequate training of health professionals in skills to deal with telehealth aiming at safe and effective care.
“In my age, we didn't have the computers”: Using a complexity lens to understand uptake of diabetes eHealth innovations into primary care-A qualitative study [41]	10 health care professionals (5 physicians, 3 nurses, and 2 dietitians)	Interviews to explore participants’ experiences with the use of a website (MyDiabetesPlan) in Canada and how it has been integrated into the clinical encounter and clinical care in diabetes. MyDiabetesPlan emphasized to clinicians a patient-centered approach that helped patients assume greater ownership for their care.	Health care professionals reported limited consultation hours and complex workflows as barriers to telehealth, identifying that senior administrative leadership (eg, medical directors and chronic disease managers), clinical reservation staff, and the information technologist were key players in reducing technological barriers and optimizing workflow-related issues.
Home telemonitoring for chronic disease management: Perceptions of users and factors influencing adoption [42]	9 clinicians	Clinicians’ experiences and perceptions on the effect of in-home telemonitoring services for chronic disease in Australia, assessed through a questionnaire and semistructured interviews. Regarding technology satisfaction and usability of the user interface in the clinical portal for scheduling patient measures and monitoring data, 56% answered that the interface was easy to use, and 56% were satisfied with telemonitoring services in the trial context.	The authors argue that the main barrier to the implementation of telehealth was acceptance by health professionals. They also argue that the lack of support from information technology technicians would be a barrier. The main challenge is to incorporate telehealth work into their existing work practice, and this includes adapting to the local organizational context, a dedicated telehealth professional, and a dedicated telehealth coordinator.
Development and validation of a mHealth technology for the promotion of self-care for adolescents with diabetes [43]	5 health professionals (clinicians) with experience in caring for adolescents diagnosed with diabetes mellitus type 1.	To develop and validate version 1.0 of the Smartphone Usability Questionnaire (SURE), a mobile app (APP-DM AGENDINHA) to promote self-care for adolescents with diabetes mellitus type 1 in Brazil. Technical validity was assessed by calculating the content validity index (CVI). A CVI value higher than 0.78 was stipulated as the desired parameter. The SURE obtained an overall content validity index of 0.96.	The authors did not discuss barriers and limitations.
Development and implementation of a decision support system to improve control of hypertension and diabetes in a resource-constrained area in Brazil: Mixed methods study [44]	96 health care professionals (25 physicians, 44 nurses, and 27 other health professionals) aged between 27 and 43 years.	Usability and satisfaction of a decision support system (web based) to improve health care and control hypertension and diabetes in an area with limited resources in Brazil. The evaluation was carried out through a questionnaire with 24 items on impressions of feasibility, usability, usefulness, and satisfaction, and had an overall Cronbach α of .93. As for usability, the general assessment was good, stating that the application was easy to understand and use. All professionals agreed that the application was useful (score of 4 or 5) to promote prevention, assist in treatment, and improve patient care. They were generally satisfied with the application (median score between 4 and 5).	The authors argue that many clinical decisions support systems (CDSSs) have not been regularly used or extensively supported by primary care physicians. The barrier cited for using CDSSs was the difficulty in running systems that are not integrated with existing health systems and health records. The authors also point to different levels of internet connectivity in primary care centers and low technological literacy among health professionals as barriers.
Rapid implementation of a diabetes telemedicine clinic during the Coronavirus Disease 2019 outbreak: Our protocol, experience, and satisfaction reports in Saudi Arabia [45]	14 health care providers who participated in the virtual clinic.	Health care providers’ satisfaction with the Diabetes Telemedicine Clinic protocol in Saudi Arabia was assessed through a satisfaction survey. Most health care providers agreed or strongly agreed that the Diabetes Telemedicine Clinic protocol is simple enough and does not require technical skills or a dedicated orientation session prior to working there (93%). The clinic almost always met its patients care treatment goals (71%), and the time spent with patients during the virtual visit was sufficient (93%).	Health professionals considered the need for technical skills with digital tools and the higher cost than usual care as barriers to telehealth. The authors cite as a limitation the difficulty in maintaining patient safety standards and protecting them against loss of privacy. The main limitations of telehealth pointed out were the inability to perform a complete physical examination and the requirement of laboratory testing that cannot be delayed.
Patient and provider perspectives on a novel mobile health intervention for low-income pregnant women with gestational or type 2 diabetes mellitus [46]	29 providers (14 physicians, 6 nurses, 2 medical assistants, 1 registered dietitian, 1 licensed clinical social worker, 1 lactation counselor, and 4 certified health educators), with the majority being female.	Feedback from health care providers regarding usability of the SweetMama prototype (a novel educational and motivational mHealth tool for pregnancy with diabetes) by interview. SweetMama was perceived to have a helpful user-friendly interface with the ability to interact with messages and the ability to select favorites.	The authors argue that health professionals considered that the characteristics of the devices, interface, and ease of use, such as functional features, visual features, and even the quality of the information, can be barriers to the adoption of digital tools. Another point discussed as a barrier was the lack of integration between platforms, with the need to switch between internal platforms and digital tools during consultations, considering that the use of digital tools took additional time for the provision of health care.
Mobile-based and cloud-based system for self-management of people with type 2 diabetes: Development and usability evaluation [47]	7 health professionals (3 physicians and 4 nurses) aged between 35 and 42 years.	The usability of a cloud-based and mobile diabetes self-management app in Iran was assessed using the User Experience Questionnaire (UEQ). The categories evaluated were attractiveness (system design), acumen (user friendly so that working with it is not time-consuming), efficiency, reliability (ability to visualize and access data online and instantly), stimulation, and novelty. The highest average was for perspicuity and the lowest was for attractiveness. This study showed that the usability of mobile and cloud-based systems could be satisfactory and promising.	The barriers discussed by the authors include the lack of technical and human resources in organizations to support the implementation of telehealth systems, the lack of financing for mobile technology solutions, the challenges of merging health care solutions with electronic health records, and other issues. The authors argue that these barriers are aggravated by the reluctance and fear of the health professional to use digital tools and the lack of sufficient time due to excess consultations.

All sampled studies were published in 2021 [37-47]. Eight of the sampled studies were conducted in high-income countries, including Australia (n=2) [39,42], Canada (n=1) [41], the United States (n=2) [37,46], Norway (n=1) [38], Portugal (n=1) [40], and Saudi Arabia (n=1) [45]. Three of the sampled studies were conducted in middle-income countries, including Brazil (n=2) [43,44] and Iran (n=1) [47]. All included studies were published in English, with one of them written in both English and Portuguese [43].

Moreover, 9 studies were qualitative studies [37,39-42,44-47], 1 was a quasiexperimental study [44], and 1 was a multisite trial [42]. Furthermore, 7 studies addressed diabetes [38,39,41,43,45-47], 1 addressed diabetes and hypertension [44], and 3 addressed chronic diseases [37,40,42]. All studies focused on primary care. Additionally, 7 studies addressed telehealth [38,40,41,43,44,46,47] and 4 addressed telemedicine [37,39,42,45] (Table 2).

**Table 2:** Relevant aspects of the included studies.

Variable			Frequency		Citation
**Level of socioeconomic development in the study country**					
	Middle-income country	8		[37-42,45,46]	
	High-income country	3		[43,44,47]	
**Study design**					
	Qualitative study	9		[37,39-42,44-47]	
	Quasiexperimental study	1		[44]	
	Multisite trial	1		[42]	
**Disease addressed in the study**					
	Diabetes	7		[38,39,41,43,45-47]	
	Diabetes and hypertension	1		[44]	
	Chronic diseases	3		[37,40,42]	
**Digital tool used for telehealth^a^**					
	Video	2		[37,45]	
	App	3		[38,43,45]	
	Smartphone	3		[39,43,46]	
	Digital platform	2		[40,42]	
	Website	1		[41]	
	Visual media	3		[37,38,45]	
	Clinical decision support system	1		[44]	
	Cloud-based mobile tool	2		[45,47]	
	Mobile health tool	1		[46]	
	Internet-based interactive support system	2		[39,45]	
	Email/WhatsApp message	1		[45]	

^a^Multiple tools have been used in individual studies.

Most of the included studies focused on the evaluation of a specific tool to deliver telehealth and were studies of the development or validation of digital tools. The telehealth tools in the included studies were apps, smartphones, websites, visual media, digital platforms, and mobile and cloud-based applications. The digital interventions described in the included studies were telemedicine [37,39,45], teleconsultation [40], telemonitoring [38,42], decision support [44], educational videos, and digital interventions for self-management [41-43,46]. Eight studies addressed usability, and 10 studies addressed satisfaction [33-41,47]. Five studies used interviews to assess usability [37,38,40-42], and 6 studies used surveys [39,43-47].

The population of the included studies covered patients and health care providers. The population of interest for our review was health care providers only. One study did not report the sample number of health care providers [39]. A total of 248 health professionals were studied. Regarding the occupation of health professionals, most studies targeted physicians or nurses as follows: 125 physicians (clinicians, medical assistants, and specialists such as cardiologists and endocrinologists), 83 nurses, 4 nutritionists, 1 occupational therapist, and 35 others not specified. All health professionals investigated in the studies had previous experience with telehealth and with the care of patients with hypertension and diabetes.

### Findings Related to the Outcomes of Interest

**Figure 2:**
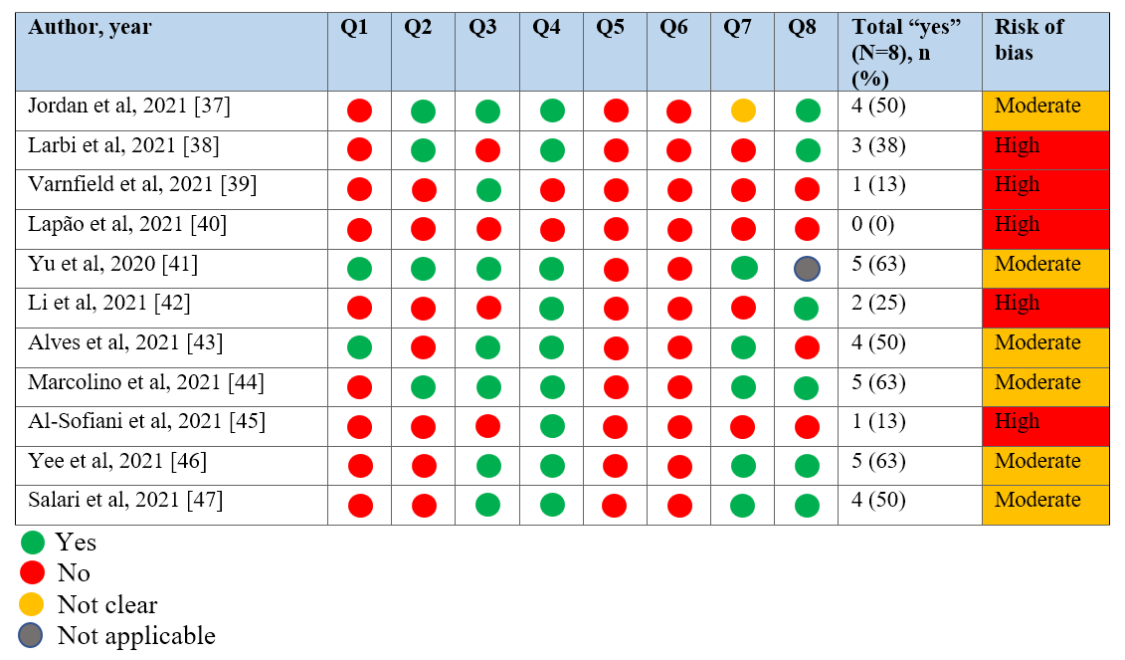
Assessment of methodological quality and risk of bias by the Joanna Briggs Institute Critical Assessment Tool for cross-sectional studies. The questions were as follows: Q1, Were the criteria for inclusion in the sample clearly defined? Q2, Were the study subjects and the setting described in detail? Q3, Was the exposure measured in a valid and reliable way? Q4, Were objective standard criteria used for measurement of the condition? Q5, Were the confounding factors identified? Q6, Were strategies to deal with confounding factors stated? Q7, Were the outcomes measured in a valid and reliable way? Q8, Was appropriate statistical analysis used?

The main finding of this review was that health professionals considered the usability of telehealth to be good and felt comfortable and satisfied using telehealth for the care of patients with hypertension and diabetes in primary care. All studies contributed to this outcome. The review set of analyses supports the assessment finding with moderate confidence (Figure 2).

Patients with hypertension and diabetes were also satisfied with using telehealth in primary care. Eight studies contributed to this finding. The analysis showed moderate confidence in this outcome.

A summary of the results is reported in Table 3. According to consensus, ease of use was the most important predictor for using digital technologies in health [37-47].

Some studies reported that the main barriers identified by health professionals were technological challenges [37,40,44,47], low computer skills [37,44,47], and a lack of appropriate equipment for telehealth [40]. In addition, the inability to perform comprehensive physical examinations [37,40,45], a lack of training [37,44], safety flaws [39,40,45], barriers to regulatory issues [37,39], scheduling difficulties [37,44], time spent scheduling new patients [37,41,44], and telehealth not integrated with other practices or members of the health care team [39,40,42] were considered. It was also reported that telehealth was not suitable when patients needed a diagnosis, procedure, or drug that could only be provided in large hospitals [37,45]; when there was a language/cultural barrier [37,41,45,46] or hearing difficulty [45]; when health professionals treated new, unstable, or undiagnosed patients [37,45]; or when the patient expressed a preference for face-to-face consultations to discuss sensitive issues [37]. Other challenges included logistics [37,40,42,44], clinical and administrative support [37,40-42], absence of a dedicated clinical care coordinator [37,40,42], connectivity issues [37,41], complexity [37,40], a lack of platform interoperability [37,40], a lack of integration between devices [37,40,42], and fear of using digital tools [37,47].

Factors that would improve the usability of the telehealth system were pointed out. These included attractive simple visual and functional features [40,41], having a provider in the room with the patient (ie, a dedicated telepresenter) [37,45], having dedicated time for telehealth [42,44], receiving standardized training [37,40,42,44], having dedicated staff to schedule and coordinate telehealth [37,39,40,42], improving security [37,39,40] and privacy criteria [37,39,40], and running systems that are interoperable and integrated into existing health systems and health records [37,39,40].

Only 1 study [40] pointed out risks with the use of telehealth, which included the possibility of incorrect diagnosis, equipment malfunction, and privacy violations.

**Table 3:** CERQual (Confidence in Evidence from Qualitative Research Reviews) summary of the qualitative findings.

Summary of review finding^a^	Studies contributing to the review finding	Methodological limitations	Coherence	Relevance	CERQual^b^ assessment of confidence in the evidence
Health professionals considered the usability of telehealth in the care of patients with hypertension and diabetes in primary care to be good. They reported benefits, mainly related to access to specialized services, improved communication, cost reduction, and improved follow-up continuity by allowing remote follow-up.	All studies contributed to this outcome.	Moderate concerns regarding methodological limitations that will probably reduce confidence in the review finding.	No or very minor concerns regarding coherence that are unlikely to reduce confidence in the review finding.	Moderate concerns regarding relevance. Only 8 countries were represented in the studies (6 high-income countries, 2 middle-income countries, and 0 low-income countries).	Moderate confidence: it is likely that the review finding is a reasonable representation of the phenomenon of interest.
Health professionals felt comfortable and satisfied using telehealth for the primary care of patients with hypertension and diabetes.	Eight articles addressed satisfaction with telehealth.	Moderate concerns regarding methodological limitations that will probably reduce confidence in the review finding.	No or very minor concerns regarding coherence that are unlikely to reduce confidence in the review finding.	Moderate concerns regarding relevance. Most studies evaluated a type of digital tool in a limited setting. Therefore, it is not clear whether the findings of the phenomenon are applicable, transferable, generalizable, or valid in all contexts.	Moderate confidence: it is likely that the review finding is a reasonable representation of the phenomenon of interest.
Patients felt satisfied with telehealth for hypertension and diabetes.	Eight articles addressed satisfaction with telehealth.	Moderate concerns regarding methodological limitations that will probably reduce confidence in the review finding.	No or very minor concerns regarding coherence that are unlikely to reduce confidence in the review finding.	Moderate concerns regarding relevance. Most studies evaluated a type of digital tool in a limited setting. Therefore, it is not clear whether the findings of the phenomenon are applicable, transferable, generalizable, or valid in all contexts.	Moderate confidence: it is likely that the review finding is a reasonable representation of the phenomenon of interest.

^a^The objective was to synthesize evidence on health professionals’ perceptions of the usability of telehealth in the primary care of individuals with noncommunicable diseases (hypertension and diabetes) from the COVID-19 pandemic onward. The study aimed to enhance our understanding of whether health professionals can use telehealth to achieve specific goals in the care of patients with hypertension and diabetes in primary care with effectiveness, efficiency, and satisfaction in a particular context of use. The types of digital tools addressed in the studies were as follows: web-based video conferencing, apps, websites, social media, smartphones, internet-based interactive support, digital monitoring services, email, WhatsApp, tablets, clinical decision support systems, mobile apps, and mobile-based and cloud-based systems.

^b^CERQual: Confidence in Evidence from Qualitative Research Reviews.

### Methodological Quality, Risk of Bias, and Limitations of the Included Studies

A considerable number of issues compromised methodological quality and increased the risk of bias in the included studies. None of the studies presented a sample size calculation, and all of them were nonprobabilistic convenience samples, with small samples, which reduced the credibility of the data representation. Most health professionals were recruited through expert sampling, which may have impaired the external validity of the studies. Besides this, all studies provided critical information necessary for detailing of the sample, with explicit inclusion and exclusion criteria for recruiting study participants. Most studies did not describe subjects and settings in detail, thus compromising external validity.

Most studies did not use a validated and reliable instrument to assess usability. In some studies, usability results from the health care providers’ perspectives were not presented in depth, which reduced the credibility of the data and impacted the accuracy of the findings.

Another recurring problem was that none of the studies addressed confounders or described strategies to analyze the influence of possible confounders on the results, which increased the risk of bias. Aspects, such as participant age, previous experience with digital tools, and whether telehealth was offered in a private/public service or a rural/urban area, can influence or confuse the results found. Developing an in-depth understanding of where telehealth is offered, by whom, and in what situation, is essential to deepen the discussion of its usability.

Regarding data adequacy, most of the included studies supported the results found (Multimedia Appendix 2). Despite the small number of participants in each study, the overall number of participants in our review did not impact our claims about the usability of telehealth from the perspective of health care professionals in the investigated contexts, even though it precluded the possibility of making broad claims about telehealth usability in any context. Figure 2 shows the classification of the risk of bias in the analyzed studies. Six studies (55%) had a moderate risk of bias and were considered to have moderate methodological quality, and 5 studies (45%) had a high risk of bias and were considered to have low methodological quality. 

### Quality of the Evidence and Strength of the Recommendations

The quality of the evidence regarding the usability of telehealth systems for the care of patients with hypertension and diabetes in primary care from the view of health professionals was considered moderate. These findings are a reasonable representation of the phenomenon of interest. Moderate concerns were found regarding methodological limitations, coherence, adequacy, and relevance, which reduced confidence in the findings. Thus, it is possible that more research in different contexts of use will influence the confidence of these findings.

## Discussion

### Principal Findings

This synthesis of qualitative evidence examined the usability of telehealth systems for patients with diabetes and hypertension in primary care from the perspective of health professionals who use digital tools. Our findings suggest that health care professionals consider the usability of telehealth systems to be good. The quality of evidence for this finding was considered moderate. With moderate confidence, we found that health care professionals felt comfortable and satisfied using telehealth to care for their patients with diabetes and hypertension. Likewise, patients with diabetes and hypertension felt satisfied being cared for using telehealth systems.

### Telemedicine and Telehealth

According to the Centers for Disease Control and Prevention (CDC), although often used interchangeably, the terms “telehealth” and “telemedicine” are not synonymous [48]. For the World Health Organization, however, there is no clear distinction between these terms [49]. Telemedicine is defined as “the use of electronic information and communications technologies to provide and support health care when distance separates the participants” [50]. A broader definition of telemedicine encompasses both clinical and nonclinical applications [50]. Clinical applications of telemedicine involve patient care, including diagnosis, treatment, and other medical decisions or services for specific patients. Nonclinical uses of telemedicine include continuing medical education and management meetings [51].

Telehealth is a large and heterogeneous collection of clinical practices, technologies, and arrangements shared with information technologies involving different strategies and tools. These may include telemedicine and various nonmedical services, such as telenutrition, telenursing, telepharmacy, teledentistry, teleaudiology, teleneuropsychology, telerehabilitation, teletrauma, tele-electrocardiography, teleradiology, telepathology, and even teleabortion in countries where this practice is allowed [4]. Telehealth can be conceptualized as the use of electronic information and telecommunication technologies to support long-distance clinical health care, health-related education of patients and professionals, public health, and health administration [5,9,12,13,52]. Technologies include video conferencing, the internet, image storage and forwarding, media streaming, and terrestrial and wireless communications [4]. The wide and heterogeneous collection of clinical practices and technologies used to offer telehealth expands the range of possibilities, but, on the other hand, it becomes a challenge to synthesize and compare them.

In our study, we differentiated between telehealth and telemedicine based on the type of service offered and the terminology used by the authors. The studies included in our review evaluated different telehealth modalities with different systems. The modalities included video telemedicine, teleconsultation, patient data monitoring, alerts, therapeutic management, and remote monitoring, and involved apps, websites, visual media, smartphones, internet-based interactive support systems, web-based decision support systems, educational videos, and mobile devices.

### Telehealth Before COVID-19

Telehealth has a long history. Telehealth systems have been used for decades in clinical settings, mainly in high- and middle-income countries [53]. In an 1879 article in the Lancet, there was talk of using the telephone to reduce unnecessary office visits. In 1906, Einthoven published an article dealing with the requirement for transtelephonic transmission of electrocardiography information from the physiology laboratory to the clinic at the Academic Hospital about a kilometer away (“télécardiogramme”) [54]. The radio has been used since the 1920s to provide medical advice to ship clinics. In 1925, a cover of Science and Invention magazine showed a doctor diagnosing a patient by radio, and the article inside it imagined a device that would allow the video examination of a patient from a distance [55]. In 1948, 24 miles apart in Pennsylvania, 2 health professionals transmitted the first radiographic images over the telephone. In Alaska, for decades, otoscopy and audiometry have been performed by community health workers in small villages, and information is sent to specialists in other cities.

As technology evolves, other digital tools are incorporated into telehealth. An example is home monitoring, which developed more fully during the Mercury space program when the National Aeronautics and Space Administration (NASA) began to perform remote physiological monitoring, and for more than 50 years, NASA’s work in telemetry, remote communication, and life sciences has led to unprecedented advances in digital health [56].

Government agencies and health care providers have turned to telehealth in response to disasters over the years. In the year 2000, the North Atlantic Treaty Alliance (NATO) (an intergovernmental military alliance between 29 members, including North American and European countries) developed a Multinational Telemedicine System that was deployed with its military forces during several crises [57]. Through solutions, such as implantable portable telemedicine personal kits and satellite linkage, areas in need received health support from medical specialists located in other countries [58]. During hurricanes Harvey and Irma, 7 private telemedicine companies provided care to victims [59]. In 2003, after the severe acute respiratory syndrome (SARS) pandemic, China began to explore telehealth and integrated electronic medical systems for use in similar situations in the future. In 2019, telehealth was offered to people affected by wildfires [59].

The usability challenges of telehealth from the health professional’s perspective and other human factors involving information technology (IT) have been less studied than usability from the patient’s point of view. However, the challenges are huge. The literature on human factors and organizational aspects of telehealth shows usability problems of telehealth systems experienced by health professionals. Examples include a potential negative impact on patient safety, poor workflow integration, frustration, and burnout when practitioners spend more time and effort integrating telehealth into their work routines [60]. Additionally, before the pandemic, some health professionals reported hating their computers, and others said they feared technology, believing that with telehealth, it would not be possible to maintain the quality of care [60].

### Telehealth From COVID-19 Onward

The widespread adoption of telehealth, which many proponents have advocated for years, is one of the most significant shifts in health care that the COVID-19 pandemic has brought. This triggered regulatory changes that lowered the barriers to telehealth in several countries worldwide, resulting in the large-scale expansion of its use [61]. Patients and health professionals found extrinsic motivations to expand telehealth, such as the possibility of avoiding face-to-face contact, reducing costs, reducing the need for travel, and providing specialized care, even in remote areas. If, before the pandemic, patients and even health professionals were intimidated by technology and questioned whether telehealth could be an efficient way to take care of health, today, most consider telehealth helpful with quality equal to or greater than that of traditional care, mainly for patients with NCDs [9]. However, most organizations and professions were not ready for telehealth before the pandemic, causing staff resistance and lack of use of this technology in the interprofessional setting when most needed [62].

Telehealth and its effectiveness have been examined for various diseases [50]. Two recent systematic reviews analyzed the effectiveness of the use of telehealth programs for the care of individuals with hypertension and diabetes mellitus, demonstrating that telehealth was an effective tool for the health care of these patients, which improved the care experience (eg, patient awareness and engagement) [63,64]. Patient satisfaction with telehealth has also been researched, showing good results [1,2,65,66]. The main contribution of this study to the field of knowledge is the addition of the perception of health professionals who adopted this strategy to care for patients with hypertension and diabetes from the COVID-19 pandemic onward.

### Usability of Telehealth for the Care of Patients With Hypertension and Diabetes

Usability can be defined as the extent to which a product can be used by specific users to achieve specific goals of effectiveness, efficiency, and satisfaction in a specific context of use [67]. The concept of usability, therefore, comprises a combination of user actions and attitudes so that the quality of the system depends on the degree to which the system satisfies the stated and implied needs of its various stakeholders, providing value. In this way, different approaches to what makes a product “usable” are divided into looking at the product with the product’s vision in mind (ie, functional, visual, and ergonomic characteristics) and looking from the end user’s point of view (ie, effectiveness, efficiency, security regarding confidentiality, and satisfaction of use), involving the ability to solve and manage problems and the ease of use of the product in the real world [68]. Evidence has associated dissatisfaction and high dropout rates in telehealth with the lack of some of these factors [20,24].

The usability of systems is one of the essential quality attributes. Good usability of telehealth is crucial for its adoption and acceptance, and the health professional’s involvement with it [59,69,70]. It is one of the most significant predictors of intended use [69]. However, there are more studies addressing usability from the patient’s perspective than the health professional’s perspective. A Cochrane Database of Systematic Reviews 2020 concluded that further studies are needed to address the perceptions and experiences of health professionals using digital tools to provide primary health services in high-, low-, and middle-income countries [22]. Our review included high-income countries and middle-income countries. However, no low-income country was included.

In several high-income countries, telehealth is already a reality. In this way, important issues, such as those related to implementing and managing change in IT in health, have already been overcome [71]. In these locations, the core IT infrastructure is in place, and health care organizations are investing in designing and implementing the next generation of health care IT “byproducts,” such as clinical dashboards, status displays, clinical decision support, and technologies geared toward the patient [71]. However, in low- and middle-income countries, telehealth challenges still include issues and costs related to implementing the technology. Furthermore, in low- and middle-income countries, rapid globalization has progressively increased the incidence of NCDs. Low- and middle-income countries have higher NCD mortality rates, whereby 77% of all NCD deaths are in low- and middle-income countries [2]. Most of these countries face problems with infrastructure and the need for patients to travel long distances to hospitals [2]. In these countries, telehealth can be particularly beneficial to expand access to health care. In these countries, the barriers and challenges of telehealth may differ from those faced by high- and middle-income countries. In this way, understanding the usability of telehealth from the perspective of the health professional who uses the tool in low-income countries can bring enormous contributions to global health and is a gap in the literature.

Usability has been evaluated with various methods and measurements over the years [18,69]. Most studies included in this review, however, evaluated usability as a quality characteristic. Few studies specified other attributes, such as security and efficiency. Additionally, we found that most studies included interviews and surveys, making reproduction and comparison between studies difficult. Only 2 studies used standardized instruments for assessment, and it was more common for studies to use author-created questionnaires for usability. Our results are somewhat in line with findings from a previous review that found that usability was primarily assessed through polls and questionnaires [69]. It is possible that the wide variety of the types of digital tools has created different assessment needs that are not captured in existing standardized usability questionnaires. Furthermore, usability may more often be a concern of computer science researchers, while the cognitive impact is a concern of researchers and health care professionals. In addition, our review revealed that telehealth usability for the care of patients with hypertension and diabetes was mainly studied by physicians and nurses. Understanding usability from the perspectives of other health professionals is relevant and important. Gender and age issues, which are known to influence telehealth usability, were also not addressed by most studies.

It is important to point out that in the usability evaluation, we investigated usability criteria exclusively. We did not evaluate the quality of the content and functions, or the effectiveness of telehealth. Furthermore, it should be mentioned that a usability assessment cannot claim to cover all possible and critical usage situations that may occur.

### Main Capabilities of Telehealth

The main positive points from the perspectives of health care professionals were that telehealth enabled the maintenance of care without the need for face-to-face contact, as well as enabled follow-up, co-management between primary care providers and specialists even in distant locations, communication between patients and health professionals, and health education. They also believed that telehealth was successful in influencing the attitudes and behaviors of patients and promoting self-management, with improvements in health conditions, cost savings, decision support, and flexibility regarding the health care system [37-44].

### Barriers to Telehealth

The biggest challenge for research in the field of telehealth is likely to be looking at obstacles and enablers for both health care professionals and patients. Low level of computer skills, lack of motivation, and little knowledge about the clinical utility of telehealth represent major cultural barriers to the routine use of telehealth, from the point of view of both the health professional and the patient.

Some providers pointed out that telehealth would not be suitable for exacerbations mainly because of the inability to perform a physical examination [37,40,45]. Limited specific IT infrastructure, including regular power supply [37,41], internet connection [37,41], gaps in interoperability [39,42,44,46,47], computer availability [37,40,44,47], and availability of specific staff for support and problem solving [37,40,44,47], were also barriers cited in the studies. However, gaps were generally larger for staff than for physical infrastructure, suggesting that, in addition to IT infrastructure, the shortage of essential personnel imposes significant constraints on the adoption of telehealth interventions. It has already been demonstrated that low technological competence [37,44,47] and an inability to perform physical examination are the main barriers cited by health professionals in adopting telehealth [37,40,45,47]. As proposed by Nouri et al [10], proactively exploring potential disparities in access to telehealth, developing solutions to mitigate barriers to digital literacy, removing barriers created by the health system for telehealth, and advocating for policies and infrastructure that facilitate equitable access to telehealth can ensure that the current implementation of telehealth does not exacerbate health disparities. Developing a strong evidence base for the use of telehealth will help to better understand how to overcome its challenges [48].

### Strengths

This systematic literature review consulted 7 research databases to control for sample bias. We accepted only peer-reviewed publications to control validity, including manual searching in Google Scholar and gray literature, which helped to reduce publication bias and to improve the internal and external validity of the study. To control design bias, the systematic review protocol was registered in PROSPERO and previously published.

### Limitations

The lack of standardization to describe usability was a key study limitation. Different authors used different terminologies to describe usability, which may have limited the inclusion of some articles in the review. Most of the studies evaluated usability through qualitative instruments, such as surveys, making it difficult to obtain objective information. These types of studies have important limitations due to the way they are constructed and the way they are interpreted by the respondents, which can cause difficulties in generalizing the data, therefore affecting its validity and reliability. Another limitation of the study was the impossibility of generalizing the results beyond the context of primary care in high- and middle-income countries, since only studies from high- and middle-income countries and from primary care were included. Future research should assess telehealth usability in low- and middle-income countries to build a broader picture of the challenges faced using telehealth across the world.

### Implications of the Results for Practice, Policy, and Future Research

This systematic review showed that health care professionals who used telehealth for the care of patients with hypertension and diabetes from the COVID-19 pandemic onward considered the usability to be good, and most studies showed that health care professionals felt comfortable and satisfied with its use. Based on this, there are some policy implications and recommendations that can be derived from this research.

Improving health care for people with NCDs is a huge challenge. This challenge includes health promotion even in situations where face-to-face contact is not possible, as was the case with the COVID-19 pandemic. People with NCDs require ongoing health care and a team of health professionals with different modalities of care, such as follow-up, monitoring, intervention, rehabilitation, education, self-management learning, and consultation with specialists. Telehealth has the potential to alleviate these challenges, even after the pandemic. Understanding the usability from the perspectives of health professionals who frequently use telehealth systems (with weaknesses and potential) can facilitate the consolidation of telehealth, including expanding to those places where access to health services for people with chronic diseases is limited, such as low- and middle-income countries.

In general, concerns about the adequacy of data regarding the usability of telehealth by health professionals did not affect our confidence in interpreting the results. However, there is a need for research that investigates other factors that may influence perceptions of telehealth usability, such as differences between private and public services; differences in the level of experience of professionals, including professional experience and experience with digital tools; and differences in gender, age groups, occupations, and community settings (eg, rural and urban areas). Questions about internet connectivity quality, interface quality for the provision of telehealth, and previous training in telehealth practice should also be investigated as they may influence the perception of usability among health professionals who use telehealth systems.

### Conclusion

Telehealth has been used for years to increase patient access to care and provide effective remote health care services. However, it was after the COVID-19 pandemic that telehealth prospered and emerged around the world as an indispensable resource for improving access to care, empowering patients, influencing their attitudes and behaviors, and ultimately enhancing their medical conditions.

Increasingly, the focus of chronic disease management is shifting to home and community settings, with telehealth quickly becoming the solution of choice. The favorable perception of professionals about telehealth systems in the treatment of NCDs points to the advancement of digital health experiences. The most significant barriers pointed out by health professionals who use telehealth to care for people with NCDs are related to empowering people for digital health. They pointed out that these barriers are greater than infrastructure barriers. The findings of this study can contribute to overcoming barriers and strengthening telehealth in the care of patients with NCDs beyond the period of the COVID-19 pandemic.

## Appendix

Multimedia Appendix 1 Detailed search strategy.

Multimedia Appendix 2 Critical Appraisal Skills Program checklist.

Multimedia Appendix 3 Excluded studies and reasons for exclusion.

## Abbreviations

CASP: Critical Appraisal Skills Program
GRADE-CERQual: Grading of Recommendations Assessment, Development and Evaluation - Confidence in Evidence from Qualitative Research Reviews
IT: information technology
JBI: Joanna Briggs Institute
NASA: National Aeronautics and Space Administration
NCD: noncommunicable disease
PEOTS: patient/population-exposure-outcome-time-study design

## Data Availability

All data generated or analyzed during this study are included in this published article and its supplementary information files.
