# Supplementary material for: Usability of Telehealth Systems for Noncommunicable Diseases in Primary Care From the COVID-19 Pandemic Onward: Systematic Review
Source: J Med Internet Res. 2023 Mar 16;25:e44209. doi: 10.2196/44209 (PMC10022651; doi:10.2196/44209)
Supplement: Multimedia Appendix 1 [file jmir_v25i1e44209_app1.docx]

**Multimedia Appendix 1.** Detailed search strategy.

**Synthesis, keywords, and search strategy performed on PubMed**

("Telemedicine"[Mesh] OR "digital health" OR telecare OR Telehealthcare OR "Telerehabilitation"[Mesh] OR "Remote Consultation"[Mesh] OR "Telemanagement") AND ("Chronic Disease"[Mesh] OR "Noncommunicable Diseases"[Mesh] OR "Hypertension"[Mesh] OR "Diabetes Mellitus"[Mesh]) AND ("Evaluation Studies as Topic"[Mesh] OR "Program Evaluation"[Mesh] OR "Evaluation Study" [Publication Type] OR "Health Care Evaluation Mechanisms"[Mesh] OR "Health Care Quality, Access, and Evaluation"[Mesh] OR assessment OR evaluation OR "user experience" OR usability OR "User-Computer Interface"[Mesh] OR "User Centered Design" OR "User-Centered Designs" OR "Usability Testing" OR "Testing, Usability")

Filter: Publication date

**4.5 Synthesis, keywords, and search strategy performed on EMBASE**

#1 (((((((((((((((((((((((((('telemedicine' OR 'telemedicine'/exp OR telemedicine OR 'decision' OR 'decision'/exp OR decision) AND making, AND 'computer assisted' OR 'decision' OR 'decision'/exp OR decision) AND making, AND 'computer assisted' OR digital) AND ('health' OR 'health'/exp OR health) OR digital) AND ('health' OR 'health'/exp OR health) OR 'telecare' OR 'telecare'/exp OR telecare OR telehealthcare OR 'telerehabilitation' OR 'telerehabilitation'/exp OR telerehabilitation OR remote) AND ('consultation' OR 'consultation'/exp OR consultation) OR remote) AND ('consultation' OR 'consultation'/exp OR consultation) OR telemanagement) AND chronic AND ('diseases' OR 'diseases'/exp OR diseases) OR chronic) AND ('diseases' OR 'diseases'/exp OR diseases) OR noncommunicable) AND ('diseases' OR 'diseases'/exp OR diseases) OR noncommunicable) AND ('diseases' OR 'diseases'/exp OR diseases) OR 'hypertension' OR 'hypertension'/exp OR hypertension OR 'diabetes' OR 'diabetes'/exp OR diabetes) AND mellitus OR 'diabetes' OR 'diabetes'/exp OR diabetes) AND mellitus AND ('health' OR 'health'/exp OR health) AND ('care' OR 'care'/exp OR care) AND ('evaluation' OR 'evaluation'/exp OR evaluation) AND mechanisms OR 'health' OR 'health'/exp OR health) AND ('care' OR 'care'/exp OR care) AND ('evaluation' OR 'evaluation'/exp OR evaluation) AND mechanisms OR 'health' OR 'health'/exp OR health) AND ('care' OR 'care'/exp OR care) AND quality, AND access, AND ('evaluation' OR 'evaluation'/exp OR evaluation) OR 'health' OR 'health'/exp OR health) AND ('care' OR 'care'/exp OR care) AND quality, AND access, AND ('evaluation' OR 'evaluation'/exp OR evaluation) OR 'assessment' OR 'assessment'/exp OR assessment OR 'evaluation' OR 'evaluation'/exp OR evaluation OR user) AND ('experience' OR 'experience'/exp OR experience) OR 'usability' OR 'usability'/exp OR usability OR 'user computer') AND ('interface' OR 'interface'/exp OR interface) OR 'user computer') AND ('interface' OR 'interface'/exp OR interface) OR 'design,' OR 'design,'/exp OR design,) AND 'user centered' OR designs,) AND 'user centered' OR 'user centered') AND ('design' OR 'design'/exp OR design) OR 'user centered') AND ('design' OR 'design'/exp OR design) OR 'user centered') AND designs OR 'usability' OR 'usability'/exp OR usability) AND ('testing' OR 'testing'/exp OR testing) OR 'usability' OR 'usability'/exp OR usability) AND ('testing' OR 'testing'/exp OR testing) OR testing,) AND ('usability' OR 'usability'/exp OR usability) AND ('human'/de OR 'teleconsultation'/de) AND (2021:py OR 2022:py) AND ('chronic disease'/dm OR 'diabetes mellitus'/dm OR 'hypertension'/dm OR 'non insulin dependent diabetes mellitus'/dm)

Filters: Publication year

Study types: Human, Teleconsultation

Diseases: Diabetes Mellitus - (425), Hypertension - (391), Coronavirus Disease 2019 - (384), non-Insulin-dependent Diabetes Mellitus- (254), Pandemic - (250), Insulin-dependent diabetes Mellitus - (136), Diabetic retinopathy - (90), Diabetic foot - (41), Diabetic Ketoacidosis - (22), Maternal Hypertension - (22), Diabetic Complication - (19)

**4.6 Synthesis, keywords, and search strategy performed on** **BIREME**

(Telemedicine) OR ("digital health") OR (telecare) OR (Telehealthcare) OR (Telerehabilitation) OR ("Remote Consultation") OR (Telemanagement) AND ("Chronic Disease") OR ("Noncommunicable Diseases") OR (Hypertension) OR ("Diabetes Mellitus" ) AND ("Evaluation Studies as Topic" ) OR ("Program Evaluation") OR ("Evaluation Study") OR ("Health Care Evaluation Mechanisms") OR ("Health Care Quality, Access, and Evaluation") OR (assessment) OR (evaluation) OR ("user experience") OR (usability) OR ("User-Computer Interface") OR ("User Centered Design") OR ("User-Centered Designs") OR ("Usability Testing") OR ("Testing, Usability")

Filters: Publication year (2020 and 2021)

**4.7 Synthesis, keywords, and search strategy performed on** **IEEE Xplore**

("All Metadata":Telemedicine OR "All Metadata":digital health OR "All Metadata":telecare OR "All Metadata":Telehealthcare OR "All Metadata":Telerehabilitation OR "All Metadata":Remote Consultation OR "All Metadata":Telemanagement) AND ("All Metadata":Chronic Disease OR "All Metadata":Noncommunicable Diseases OR "All Metadata":Hypertension OR "All Metadata":Diabetes Mellitus) AND ("All Metadata":Evaluation Studies as Topic OR "All Metadata":Program Evaluation OR "All Metadata":Evaluation Study OR "All Metadata":Health Care Evaluation Mechanisms OR "All Metadata":Health Care Quality, Access, and Evaluation OR "All Metadata":assessment OR "All Metadata":evaluation OR "All Metadata":user experience OR "All Metadata":usability OR "All Metadata":User-Computer Interface OR "All Metadata":User Centered Design OR "All Metadata":User-Centered Designs OR "All Metadata":Usability Testing OR "All Metadata":Testing, Usability)

JournalsMagazinesEarly Access Articles

**Filters Applied:**JournalsEarly Access Articles, Diseases, patient monitoringhealth caremedical computingtelemedicinepatient diagnosispatient rehabilitation, 2020 - 2021

**4.9 Synthesis, keywords, and search strategy performed on BVS**

((telemedicine) OR ("digital health") OR (telecare) OR (telehealthcare) OR (telerehabilitation) OR ("Remote Consultation") OR (telemanagement) AND ("Chronic Disease") OR ("Noncommunicable Diseases") OR (hypertension) OR ("Diabetes Mellitus" ) AND ("Evaluation Studies as Topic" ) OR ("Program Evaluation") OR ("Evaluation Study") OR ("Health Care Evaluation Mechanisms") OR ("Health Care Quality, Access, and Evaluation") OR (assessment) OR (evaluation) OR ("user experience") OR (usability) OR ("User-Computer Interface") OR ("User Centered Design") OR ("User-Centered Designs") OR ("Usability Testing") OR ("Testing, Usability")) AND (type_of_study:("clinical_trials" OR "diagnostic_studies" OR "evaluation_studies" OR "guideline" OR "health_economic_evaluation" OR "prognostic_studies" OR "qualitative_research") AND la:("en"))

Filters: Publication year (2020 and 2021)

**4.10** **Synthesis, keywords, and search strategy performed on gray literature**

[**http://www.greylit.org/library/search**](http://www.greylit.org/library/search)

(Telemedicine) OR ("digital health") OR (telecare) OR (Telehealthcare) OR (Telerehabilitation) OR ("Remote Consultation") OR (Telemanagement) AND ("Chronic Disease") OR ("Noncommunicable Diseases") OR (Hypertension) OR ("Diabetes Mellitus" ) AND ("Evaluation Studies as Topic" ) OR ("Program Evaluation") OR ("Evaluation Study") OR ("Health Care Evaluation Mechanisms") OR ("Health Care Quality, Access, and Evaluation") OR (assessment) OR (evaluation) OR ("user experience") OR (usability) OR ("User-Computer Interface") OR ("User Centered Design") OR ("User-Centered Designs") OR ("Usability Testing") OR ("Testing, Usability") AND (Telemedicine) OR ("digital health") OR (telecare) OR (Telehealthcare) OR (Telerehabilitation) OR ("Remote Consultation") OR (Telemanagement) AND ("Chronic Disease") OR ("Noncommunicable Diseases") OR (Hypertension) OR ("Diabetes Mellitus") AND ("Evaluation Studies as Topic") OR ("Program Evaluation") OR ("Evaluation Study") OR ("Health Care Evaluation Mechanisms") OR ("Health Care Quality, Access, and Evaluation") OR (assessment) OR (evaluation) OR ("user experience") OR (usability) OR ("User-Computer Interface") OR ("User Centered Design") OR ("User-Centered Designs") OR ("Usability Testing") OR ("Testing, Usability")

www.mhealthevidence.org

mHealth Knowledge: mhealthknowledge.org

**4.11 Synthesis, keywords, and search strategy performed on manual search**

Searches in Google Scholar: scholar.google.co.za).

((telemedicine) OR ("digital health") OR (telecare) OR (telehealthcare) OR (telerehabilitation) OR ("Remote Consultation") OR (telemanagement) AND ("Chronic Disease") OR ("Noncommunicable Diseases") OR (hypertension) OR ("Diabetes Mellitus" ) AND ("Evaluation Studies as Topic" ) OR ("Program Evaluation") OR ("Evaluation Study") OR ("Health Care Evaluation Mechanisms") OR ("Health Care Quality, Access, and Evaluation") OR (assessment) OR (evaluation) OR ("user experience") OR (usability) OR ("User-Computer Interface") OR ("User Centered Design") OR ("User-Centered Designs") OR ("Usability Testing") OR ("Testing, Usability")) AND (type_of_study:("clinical_trials" OR "diagnostic_studies" OR "evaluation_studies" OR "guideline" OR "health_economic_evaluation" OR "prognostic_studies" OR "qualitative_research") AND la:("en"))

Filters: Publication year (2020 and 2021)
