# Supplementary material for: Usability of Telehealth Systems for Noncommunicable Diseases in Primary Care From the COVID-19 Pandemic Onward: Systematic Review
Source: J Med Internet Res. 2023 Mar 16;25:e44209. doi: 10.2196/44209 (PMC10022651; doi:10.2196/44209)
Supplement: Multimedia Appendix 2 [file jmir_v25i1e44209_app2.docx]

**Multimedia Appendix 2.** Critical Appraisal Skills Program checklist.

Paper for appraisal and reference:

- **Jordan DN, Jessen CM, Ferucci ED. Views of Patients and Providers on the Use of Telemedicine for Chronic Disease Specialty Care in the Alaska Native Population. Telemed J E Health. 2021 Jan;27(1):82-89. doi: 10.1089/tmj.2019.0284. Epub 2020 Apr 13. PMID: 32286156.**

Section A: Are the results valid? yes

1. **Was there a clear statement of the aims of the research?**

HINT: Consider

• what was the goal of the research

• why it was thought important

• its relevance

| x |
| --- |
|  |
|  |

Yes

Can’t Tell

No

Comments:

40% feel comfortable using telemedicine for chronic illness, 30% feel very comfortable, 10% neutral, 20% somewhat uncomfortable, and no one feels very uncomfortable.

**- Contexts in which the mobile devices are used:** Used for telemedicine in primary care in remote, rural Alaska.

**Limitations:** small convenience sample with more experienced professionals. This can cause bias.

1. **Is a qualitative methodology appropriate?**

| x |
| --- |
|  |
|  |

Yes

HINT: Consider

• If the research seeks to interpret or illuminate the actions and/or subjective experiences of research participants

• Is qualitative research the right methodology for addressing the research goal

Can’t Tell

No

Is it worth continuing? No

1. **Was the research design appropriate to address the aims of the research?**

| x |
| --- |
|  |
|  |

Yes

HINT: Consider

• if the researcher has justified the research design (e.g. have they discussed how they decided which method to use)

Can’t Tell

No

4 **- Was the recruitment strategy appropriate to the aims of the research?**

HINT: Consider

• If the researcher has explained how the participants were selected

• If they explained why the participants, they selected were the most appropriate to provide access to the type of knowledge sought by the study

• If there are any discussions around recruitment (e.g. why some people chose not to take part)

|  |
| --- |
|  |
| x |

Yes

Can’t Tell

No

Comments: Provider recruitment was also conducted through purposive sampling.

5- **Was the data collected in a way that addressed the research issue?**

| x |
| --- |
|  |
|  |

Yes

HINT: Consider

• If the setting for the data collection was justified

• If it is clear how data were collected (e.g. focus group, semi-structured interview etc.)

• If the researcher has justified the methods chosen

• If the researcher has made the methods explicit (e.g. for interview method, is there an indication of how interviews are conducted, or did they use a topic guide)

• If methods were modified during the study. If so, has the researcher explained how and why

• If the form of data is clear (e.g. tape recordings, video material, notes etc.)

• If the researcher has discussed saturation of data

Can’t Tell

No

6- **Has the relationship between researcher and participants been adequately considered?**

HINT: Consider

• If the researcher critically examined their own role, potential bias (it was not discussed and influence during (a) formulation of the research questions (b) data collection, including sample recruitment and choice of location

• How the researcher responded to events during the study and whether they considered the implications of any changes in the research design

|  |
| --- |
|  |
| **x** |

**Yes**

**Can’t Tell**

**No**

7- **Have ethical issues been taken into consideration?**

HINT: Consider

• If there are sufficient details of how the research was explained to participants for the reader to assess whether ethical standards were maintained

• If the researcher has discussed issues raised by the study (e.g. issues around informed consent or confidentiality or how they have handled the effects of the study on the participants during and after the study)

• If approval has been sought from the ethics committee

| **x** |
| --- |
|  |
|  |

**Yes**

**Can’t Tell**

**No**

Comments: The Alaska Area Institutional Review Board (AAIRB) approved this project. The study was approved by the Alaska Native Tribal Health Consortium (ANTHC) and Southcentral Foundation (SCF), the participating tribal health organizations.

8- **Was the data analysis sufficiently rigorous?**

HINT: Consider

• If there is an in-depth description of the analysis process • If thematic analysis is used. If so, is it clear how the categories/themes were derived from the data

• Whether the researcher explains how the data presented were selected from the original sample to demonstrate the analysis process

• If sufficient data are presented to support the findings

• To what extent contradictory data are taken into account • Whether the researcher critically examined their own role, potential bias and influence during analysis and selection of data for presentation

|  |
| --- |
|  |
| **x** |

**Yes**

**Can’t Tell**

**No**

Comments: the description of the analysis was insufficient.

9**- Is there a clear statement of findings?**

HINT: Consider

• • If the findings are explicit

• If there is adequate discussion of the evidence both for and against the researcher’s arguments

• If the researcher has discussed the credibility of their findings (e.g. triangulation, respondent validation, more than one analyst)

• If the findings are discussed in relation to the original research question

|  |
| --- |
|  |
| **x** |

**Yes**

**Can’t Tell**

**No**

Section C: Will the results help locally? yes

10- **How valuable is the research?**

HINT: Consider

• If the researcher discusses the contribution the study makes to existing knowledge or understanding (e.g. do they consider the findings in relation to current practice or policy, or relevant research based literature

• If they identify new areas where research is necessary

• If the researchers have discussed whether or how the findings can be transferred to other populations or considered other ways the research may be used

| **x** |
| --- |
|  |
|  |

**Yes**

**Can’t Tell**

**No**

Paper for appraisal and reference:

- **Larbi D, Randine P, Årsand E, Bradway M, Antypas K, Gabarron E. Criteria for Assessing and Recommending Digital Diabetes Tools: A Delphi Study. Stud Health Technol Inform. 2021 May 27;281:850-854. doi: 10.3233/SHTI210299. PMID: 34042794**

Section A: Are the results valid? yes

1. **Was there a clear statement of the aims of the research?**

HINT: Consider

• If there are sufficient details of how the research was explained to participants for the reader to assess whether ethical standards were maintained.

• If the researcher has discussed issues raised by the study (e.g. issues around informed consent or confidentiality or how they have handled the effects of the study on the participants during and after the study).

• If approval has been sought from the ethics committee.

|  |
| --- |
|  |
| **x** |

**Yes**

**Can’t Tell**

**No**

Comments: Healthcare professionals with diabetes care experience.

Fifteen healthcare professionals with an average of 19 years’ diabetes care experience were enrolled: 20% (3/15) were medical doctors, 67% (10/15) were diabetes nurses and the remaining two were a clinical dietitian and an occupational therapist. Females made up 73% (11/15) of the panellists. The average level of experience and observed use of digital diabetes tools, on a scale from 0 - very low to 10 - very high, in the panellists’ daily practices were 6.8 and 6.5, respectively.

**Primary outcome**

with an average rating of 8.9, were Usability and Automated data recording for apps. With an average rating of 7.2, Usability, Feasibility and Acceptability were the third highest rated criteria for social media, Usability (7.9) for websites and Clinical impact (8.8) for apps.

For assessing digital diabetes self-management tools, Usability was the highest rated criterion for apps, websites, and social media with an average rating of 9.6, 8.6 and 7.4, respectively.

It was not very clear, it was not very detailed whether there was enough detail on how the research was explained to the participants for the reader to assess whether ethical standards were maintained. • It was not addressed if the researcher discussed issues raised by the study (eg, questions about informed consent or confidentiality or how they dealt with the effects of the study on participants during and after the study).

**- Satisfaction (acceptance)**

App7.2, website 7.5 social media 8.1

**Limitations:** small convenience sample with more experienced professionals. This can cause bias.

**2-Is a qualitative methodology appropriate?**

|  |
| --- |
|  |
| x |

Yes

HINT: Consider

• If the research seeks to interpret or illuminate the actions and/or subjective experiences of research participants

• Is qualitative research the right methodology for addressing the research goal

Can’t Tell

No

Is it worth continuing? No

**3- Was the research design appropriate to address the aims of the research?**

| x |
| --- |
|  |
|  |

Yes

HINT: Consider

• if the researcher has justified the research design (e.g. have they discussed how they decided which method to use)

Can’t Tell

No

Comments: The study objective was to reach a consensus on the criteria required to access and recommend digital diabetes self-management tools (apps, websites, and social media) targeting the Norwegian diabetes population.

4 **- Was the recruitment strategy appropriate to the aims of the research?**

HINT: Consider

• If the researcher has explained how the participants were selected

• If they explained why the participants they selected were the most appropriate to provide access to the type of knowledge sought by the study

• If there are any discussions around recruitment (e.g. why some people chose not to take part)

|  |
| --- |
|  |
| x |

Yes

Can’t Tell

No

Comments: Healthcare professionals working within diabetes care from the four health regions in Norway were recruited to participate as panellists in a three-round Delphi study over six weeks (May to June 2020). The authors do not provide additional information.

Though the panellists’ in this study were healthcare professionals with diabetes care experience, they might not represent the perspectives of the average healthcare person supporting patients in the use of digital diabetes tools. Panellists were asked to refer to a specific list of criteria to assess digital diabetes self-management tools targeting the Norwegian population, therefore, the consensus may not apply to other countries or health conditions.

5- **Was the data collected in a way that addressed the research issue?**

HINT: Consider

• If the setting for the data collection was justified

• If it is clear how data were collected (e.g. focus group, semi-structured interview etc.)

• If the researcher has justified the methods chosen

• If the researcher has made the methods explicit (e.g. for interview method, is there an indication of how interviews are conducted, or did they use a topic guide)

• If methods were modified during the study. If so, has the researcher explained how and why

• If the form of data is clear (e.g. tape recordings, video material, notes etc.)

• If the researcher has discussed saturation of data

| x |
| --- |
|  |
|  |

Yes

Can’t Tell

No

Comments: A Delphi study is an appropriate method for obtaining academic and experience-based consensus on identified evaluation criteria, as it elicits individualized but group-generated information and allows for a wide geographical access to experts.

6- **Has the relationship between researcher and participants been adequately considered?**

HINT: Consider

• If the researcher critically examined their own role, potential bias and influence during (a) formulation of the research questions (b) data collection, including sample recruitment and choice of location

• How the researcher responded to events during the study and whether they considered the implications of any changes in the research design

|  |
| --- |
|  |
| **x** |

**Yes**

**Can’t Tell**

**No**

Comments: It was not discussed whether the researcher critically examined his own role, potential bias and influence during [não foi discutido] (a) formulation of the research questions.

7- **Have ethical issues been taken into consideration?**

HINT: Consider

• If there are sufficient details of how the research was explained to participants for the reader to assess whether ethical standards were maintained.

• If the researcher has discussed issues raised by the study (e.g. issues around informed consent or confidentiality or how they have handled the effects of the study on the participants during and after the study)

• If approval has been sought from the ethics committee

|  |
| --- |
|  |
| **x** |

**Yes**

**Can’t Tell**

**No**

Comments: The treatment of personal information in this study was approved by the Data Protection Officer at the University Hospital of North Norway (ref. 2018/3325).

There are not enough details of how the research was explained to the participants for the reader to assess whether ethical standards were maintained.

8- **Was the data analysis sufficiently rigorous**

HINT: Consider

• If there is an in-depth description of the analysis process • If thematic analysis is used. If so, is it clear how the categories/themes were derived from the data

• Whether the researcher explains how the data presented were selected from the original sample to demonstrate the analysis process

• If sufficient data are presented to support the findings

• To what extent contradictory data are taken into account • Whether the researcher critically examined their own role, potential bias and influence during analysis and selection of data for presentation

|  |
| --- |
| **x** |
|  |

**Yes**

**Can’t Tell**

**No**

Comments:

In the first round, panellists rated these criteria on a scale from 0 - not important to 10 - extremely important, with the option to provide comments. Their ratings and comments were analysed and used for the second round. The criteria were then presented together with the average rating and each participant’s previous value for each criterion. The third round provided the panellists with another opportunity to change their opinion. The analysis of the participants’ data was done using SPSS version 25. NVivo 12 Pro was used to organize and perform an inductive thematic analysis of the qualitative data.

9**- Is there a clear statement of findings?**

HINT: Consider

• • If the findings are explicit

• If there is adequate discussion of the evidence both for and against the researcher’s arguments

• If the researcher has discussed the credibility of their findings (e.g. triangulation, respondent validation, more than one analyst)

• If the findings are discussed in relation to the original research question

|  |
| --- |
|  |
| **x** |

**Yes**

**Can’t Tell**

**No**

Comments: There is no adequate discussion of the evidence either for or against the researcher's arguments.

Section C: Will the results help locally? yes

10- **How valuable is the research?**

HINT: Consider

• If the researcher discusses the contribution the study makes to existing knowledge or understanding (e.g. do they consider the findings in relation to current practice or policy, or relevant research based literature

• If they identify new areas where research is necessary

• If the researchers have discussed whether or how the findings can be transferred to other populations or considered other ways the research may be used

| **x** |
| --- |
|  |
|  |

**Yes**

**Can’t Tell**

**No**

- **Varnfield M, Redd C, Stoney RM, Higgins L, Scolari N, Warwick R, Iedema J, Rundle J, Dutton W. M♡THer, an mHealth System to Support Women with Gestational Diabetes Mellitus: Feasibility and Acceptability Study. Diabetes Technol Ther. 2021 May;23(5):358-366. doi: 10.1089/dia.2020.0509. Epub 2020 Dec 15. PMID: 33210954; PMCID: PMC8080933**

Section A: Are the results valid? yes

1-Was there a clear statement of the aims of the research?

| x |
| --- |
|  |
|  |

Yes

Can’t Tell

No

Comments:

**Primary outcome**

All clinicians either strongly agreed or agreed that the MvTHer app improved their efficiency in caring for their patients. Ease of use is one of the most significant predictors of intention-to-use smartphone health technologies and these revisions maximized acceptability.

**- Satisfaction (acceptance)**

Our results in this study suggest that involving clinicians both in the initial design and ongoing development led to high levels of satisfaction and uptake among the health care team.

**- Contexts in which the mobile devices are used**

The MvTHer platform is the first intervention in Australia to implement mobile technology to augment face-to-face clinical care for women with a first-time diagnosis of GDM. However, even with support mechanisms in place, interoperability with existing hospital practices and information technology systems (including data privacy regulations) must be prioritized from the very earliest stage of development. This need for planning is especially salient in the unequal speed at which technology advances, relative to slower moving regulatory reform.

**- Satisfaction (acceptance)**

App7.2, website 7.5 social media 8.1

**Limitations:** small convenience sample with more experienced professionals. This can cause bias.

**2-Is a qualitative methodology appropriate?**

| x |
| --- |
|  |
|  |

Yes

HINT: Consider

• If the research seeks to interpret or illuminate the actions and/or subjective experiences of research participants

• Is qualitative research the right methodology for addressing the research goal

Can’t Tell

No

Is it worth continuing? No

**3- Was the research design appropriate to address the aims of the research?**

| x |
| --- |
|  |
|  |

Yes

HINT: Consider

• if the researcher has justified the research design (e.g. have they discussed how they decided which method to use)

Can’t Tell

No

Comments: The aim of the current study was to evaluate adoption (use and user satisfaction), multidisciplinary care coordination, and health care utilization through use of the mHealth platform, called MvTHer.

4 **- Was the recruitment strategy appropriate to the aims of the research?**

HINT: Consider

• If the researcher has explained how the participants were selected

• If they explained why the participants they selected were the most appropriate to provide access to the type of knowledge sought by the study

• If there are any discussions around recruitment (e.g. why some people chose not to take part)

|  |
| --- |
|  |
| x |

Yes

Can’t Tell

No

Comments:

2018 occurred at Redland Hospital with a goal of recruiting n = 40 women with a first-time diagnosis of GDM. Inclusion criteria included a confirmed oral glucose tolerance test diagnosis of GDM between 24 and 28 weeks of gestation, at least 16 years of age, owning and the ability to use a smart mobile phone, and the ability to speak and understand English. Exclusion criteria included women who had any other type of diabetes, severe comorbidities that would limit participation, or a known history of major psychiatric illness.

Regarding the sample of health professionals. the only information was that they were the clinicians of the patients. They did not report the number of physicians.

5- **Was the data collected in a way that addressed the research issue?**

| x |
| --- |
|  |
|  |

Yes

HINT: Consider

• If the setting for the data collection was justified

• If it is clear how data were collected (e.g. focus group, semi-structured interview etc.)

• If the researcher has justified the methods chosen

• If the researcher has made the methods explicit (e.g. for interview method, is there an indication of how interviews are conducted, or did they use a topic guide)

• If methods were modified during the study. If so, has the researcher explained how and why

• If the form of data is clear (e.g. tape recordings, video material, notes etc.)

• If the researcher has discussed saturation of data

Can’t Tell

No

6- **Has the relationship between researcher and participants been adequately considered?**

HINT: Consider

• If the researcher critically examined their own role, potential bias and influence during (a) formulation of the research questions (b) data collection, including sample recruitment and choice of location

• How the researcher responded to events during the study and whether they considered the implications of any changes in the research design

|  |
| --- |
|  |
| **x** |

**Yes**

**Can’t Tell**

**No**

Comments: The researcher did not critically examine his own role, potential bias, and influence during (a) formulation of the research questions (b) data collection, including sample recruitment and site selection.

7- **Have ethical issues been taken into consideration?**

HINT: Consider

• If there are sufficient details of how the research was explained to participants for the reader to assess whether ethical standards were maintained

• If the researcher has discussed issues raised by the study (e.g. issues around informed consent or confidentiality or how they have handled the effects of the study on the participants during and after the study)

• If approval has been sought from the ethics committee

| **x** |
| --- |
|  |
|  |

**Yes**

**Can’t Tell**

**No**

Comments:

Ethical considerations specific to women who are pregnant were followed during study conception and design as outlined by the National Statement on Ethical Conduct in Human Research 2015.12 All study procedures were approved by the CSIRO Medical Research Ethics Committee (#2020_047_RR), and Metro South Hospital and Health Service (HREC/16/QPAH/785) before enrollment commenced.

8- **Was the data analysis sufficiently rigorous?**

HINT: Consider

• If there is an in-depth description of the analysis process • If thematic analysis is used. If so, is it clear how the categories/themes were derived from the data

• Whether the researcher explains how the data presented were selected from the original sample to demonstrate the analysis process

• If sufficient data are presented to support the findings

• To what extent contradictory data are taken into account • Whether the researcher critically examined their own role, potential bias and influence during analysis and selection of data for presentation

|  |
| --- |
|  |
| **x** |

**Yes**

**Can’t Tell**

**No**

Comments:

This study had some limitations, including a relatively small sample size recruited from a single hospital, limiting the generalizability of the findings. In addition, recruitment and use of the MvTHer app and clinician portal were driven by a group of engaged clinical champions, somewhat mitigating integration and interoperability challenges inherent to the broader health care system.

9**- Is there a clear statement of findings?**

HINT: Consider

• • If the findings are explicit

• If there is adequate discussion of the evidence both for and against the researcher’s arguments

• If the researcher has discussed the credibility of their findings (e.g. triangulation, respondent validation, more than one analyst)

• If the findings are discussed in relation to the original research question

|  |
| --- |
|  |
| **x** |

**Yes**

**Can’t Tell**

**No**

Comments: It is not detailed whether the results are discussed in relation to the original research question.

Section C: Will the results help locally? yes

10- **How valuable is the research?**

HINT: Consider

• If the researcher discusses the contribution the study makes to existing knowledge or understanding (e.g. do they consider the findings in relation to current practice or policy, or relevant research based literature

• If they identify new areas where research is necessary

• If the researchers have discussed whether or how the findings can be transferred to other populations or considered other ways the research may be used

| **x** |
| --- |
|  |
|  |

**Yes**

**Can’t Tell**

**No**

- **Lapão LV, Peyroteo M, Maia M, et al. Implementation of Digital Monitoring Services During the COVID-19 Pandemic for Patients With Chronic Diseases: Design Science Approach. *J Med Internet Res*. 2021;23(8):e24181. Published 2021 Aug 26. doi:10.2196/24181**

Section A: Are the results valid? yes

1-Was there a clear statement of the aims of the research?

| x |
| --- |
|  |
|  |

Yes

Can’t Tell

No

Comments:

The aim was to share efforts and experiences in creating a digital health service during a public health emergency (ie, in the midst of a pandemic).

Usefulness of the digital platform (web-based app for teleconsultations, monitoring of patient data, alerts, and therapeutic management) for chronic diseases assessed through an interview.

**Limitations:** small convenience sample with more experienced professionals. This can cause bias.

**2-Is a qualitative methodology appropriate?**

| x |
| --- |
|  |
|  |

Yes

HINT: Consider

• If the research seeks to interpret or illuminate the actions and/or subjective experiences of research participants

• Is qualitative research the right methodology for addressing the research goal

Can’t Tell

No

Is it worth continuing? No

**3- Was the research design appropriate to address the aims of the research?**

| x |
| --- |
|  |
|  |

Yes

HINT: Consider

• if the researcher has justified the research design (e.g. have they discussed how they decided which method to use)

Can’t Tell

No

Comments:

A set of interviews with general practitioners, nurses, and clinical secretaries helped identify both the critical chronic care processes (eg, consultations frequency and service levels depending on health risks) and the major communication problems between chronic patients and health care professionals (eg, the role of each health professional, how to clearly communicate therapies, and how to improve adhesion), and moreover, in the pandemic, how to access to chronic patients at home.

We followed the Design Science Research Methodology, which is based on a process with 6 sequential steps. Design Science Research Methodology benefits from supporting the design of an artefact (the primary health care digital platform) to solve the identified problem of lacking the access to chronic patients.

4 **- Was the recruitment strategy appropriate to the aims of the research?**

HINT: Consider

• If the researcher has explained how the participants were selected

• If they explained why the participants they selected were the most appropriate to provide access to the type of knowledge sought by the study

• If there are any discussions around recruitment (e.g. why some people chose not to take part)

|  |
| --- |
|  |
| x |

Yes

Can’t Tell

No

Comments: Physicians and nurses in these 3 practices who were willing to participate in the study. Convenience non-probabilistic sampling.

This study had several limitations. Most notably, the results may not be generalizable, as only 3 health units participated in the study, and it was developed under the restrictions of COVID-19 pandemic (eg, several meetings with health professionals were conducted via videoconference).

5- **Was the data collected in a way that addressed the research issue?**

| x |
| --- |
|  |
|  |

Yes

HINT: Consider

• If the setting for the data collection was justified

• If it is clear how data were collected (e.g. focus group, semi-structured interview etc.)

• If the researcher has justified the methods chosen

• If the researcher has made the methods explicit (e.g. for interview method, is there an indication of how interviews are conducted, or did they use a topic guide)

• If methods were modified during the study. If so, has the researcher explained how and why

• If the form of data is clear (e.g. tape recordings, video material, notes etc.)

• If the researcher has discussed saturation of data

Can’t Tell

No

6- **Has the relationship between researcher and participants been adequately considered?**

HINT: Consider

• If the researcher critically examined their own role, potential bias and influence during (a) formulation of the research questions (b) data collection, including sample recruitment and choice of location

• How the researcher responded to events during the study and whether they considered the implications of any changes in the research design

|  |
| --- |
|  |
| **x** |

**Yes**

**Can’t Tell**

**No**

Comments: The researchers do not address bias and how they might have dealt with them, had they existed.

7- **Have ethical issues been taken into consideration?**

HINT: Consider

• If there are sufficient details of how the research was explained to participants for the reader to assess whether ethical standards were maintained

• If the researcher has discussed issues raised by the study (e.g. issues around informed consent or confidentiality or how they have handled the effects of the study on the participants during and after the study)

• If approval has been sought from the ethics committee

| **x** |
| --- |
|  |
|  |

**Yes**

**Can’t Tell**

**No**

Comments: The project received Ethics approval from the Lisbon Health Region Ethics Commission, and in accordance with international security (ISO 27001) and privacy (General Data Protection Regulation) standards.

8- **Was the data analysis sufficiently rigorous?**

HINT: Consider

• If there is an in-depth description of the analysis process • If thematic analysis is used. If so, is it clear how the categories/themes were derived from the data

• Whether the researcher explains how the data presented were selected from the original sample to demonstrate the analysis process

• If sufficient data are presented to support the findings

• To what extent contradictory data are taken into account • Whether the researcher critically examined their own role, potential bias and influence during analysis and selection of data for presentation

|  |
| --- |
|  |
| **x** |

**Yes**

**Can’t Tell**

**No**

9**- Is there a clear statement of findings?**

HINT: Consider

• • If the findings are explicit

• If there is adequate discussion of the evidence both for and against the researcher’s arguments

• If the researcher has discussed the credibility of their findings (e.g. triangulation, respondent validation, more than one analyst)

• If the findings are discussed in relation to the original research question

| **x** |
| --- |
|  |
|  |

**Yes**

**Can’t Tell**

**No**

Section C: Will the results help locally? yes

10- **How valuable is the research?**

HINT: Consider

• If the researcher discusses the contribution the study makes to existing knowledge or understanding (e.g. do they consider the findings in relation to current practice or policy, or relevant research based literature

• If they identify new areas where research is necessary

• If the researchers have discussed whether or how the findings can be transferred to other populations or considered other ways the research may be used

| **x** |
| --- |
|  |
|  |

**Yes**

**Can’t Tell**

**No**

- **Yu CH, McCann M, Sale J. "In my age, we didn't have the computers": Using a complexity lens to understand uptake of diabetes eHealth innovations into primary care-A qualitative study. PLoS One. 2021 Jul 7;16(7):e0254157. doi: 10.1371/journal.pone.0254157. PMID: 34234368; PMCID: PMC8263251**

Section A: Are the results valid? yes

1-Was there a clear statement of the aims of the research?

| x |
| --- |
|  |
|  |

Yes

Can’t Tell

No

Comments: The objective of this study was to explore the integration of MyDiabetesPlan into clinical care, through a complexity science lens, to identify opportunities for sense-making and self-organization that can be leveraged for scale-up and sustainability.

**Limitations:** small convenience sample with more experienced professionals. This can cause bias.

**2-Is a qualitative methodology appropriate?**

| x |
| --- |
|  |
|  |

Yes

HINT: Consider

• If the research seeks to interpret or illuminate the actions and/or subjective experiences of research participants

• Is qualitative research the right methodology for addressing the research goal

Can’t Tell

No

Is it worth continuing? No

**3- Was the research design appropriate to address the aims of the research?**

| x |
| --- |
|  |
|  |

Yes

HINT: Consider

• if the researcher has justified the research design (e.g. have they discussed how they decided which method to use)

Can’t Tell

No

Comments: Interviews to explore participants' experiences with the use of a website (MyDiabetesPlan), and how it has been integrated into the clinical encounter and clinical care in Diabetes.

4 **- Was the recruitment strategy appropriate to the aims of the research?**

HINT: Consider

• If the researcher has explained how the participants were selected

• If they explained why the participants they selected were the most appropriate to provide access to the type of knowledge sought by the study

• If there are any discussions around recruitment (e.g. why some people chose not to take part)

|  |
| --- |
|  |
| x |

Yes

Can’t Tell

No

Comments:

Following completion of this trial, we invited all clinicians (n = 29) in the five intervention sites to participate in 60-minute individual in-depth interviews.

Convenience non-probabilistic sampling. Study limitations include small sample size.

5- **Was the data collected in a way that addressed the research issue?**

| x |
| --- |
|  |
|  |

Yes

HINT: Consider

• If the setting for the data collection was justified

• If it is clear how data were collected (e.g. focus group, semi-structured interview etc.)

• If the researcher has justified the methods chosen

• If the researcher has made the methods explicit (e.g. for interview method, is there an indication of how interviews are conducted, or did they use a topic guide)

• If methods were modified during the study. If so, has the researcher explained how and why

• If the form of data is clear (e.g. tape recordings, video material, notes etc.)

• If the researcher has discussed saturation of data

Can’t Tell

No

6- **Has the relationship between researcher and participants been adequately considered?**

HINT: Consider

• If the researcher critically examined their own role, potential bias and influence during (a) formulation of the research questions (b) data collection, including sample recruitment and choice of location

• How the researcher responded to events during the study and whether they considered the implications of any changes in the research design

|  |
| --- |
|  |
| **x** |

**Yes**

**Can’t Tell**

**No**

7- **Have ethical issues been taken into consideration?**

HINT: Consider

• If there are sufficient details of how the research was explained to participants for the reader to assess whether ethical standards were maintained

• If the researcher has discussed issues raised by the study (e.g. issues around informed consent or confidentiality or how they have handled the effects of the study on the participants during and after the study)

• If approval has been sought from the ethics committee

| **x** |
| --- |
|  |
|  |

**Yes**

**Can’t Tell**

**No**

Comments:

The study was approved by the Research Ethics Boards of Markham Stouffville Hospital (CIHR protocol, v2, January 2016), North York General Hospital (13–0265), St. Michael’s Hospital (13–014), University Health Network (16–6044), and Women’s College Hospital (2014-0043-B).

8- **Was the data analysis sufficiently rigorous?**

HINT: Consider

• If there is an in-depth description of the analysis process • If thematic analysis is used. If so, is it clear how the categories/themes were derived from the data

• Whether the researcher explains how the data presented were selected from the original sample to demonstrate the analysis process

• If sufficient data are presented to support the findings

• To what extent contradictory data are taken into account • Whether the researcher critically examined their own role, potential bias and influence during analysis and selection of data for presentation

| **x** |
| --- |
|  |
|  |

**Yes**

**Can’t Tell**

**No**

9**- Is there a clear statement of findings?**

HINT: Consider

• • If the findings are explicit

• If there is adequate discussion of the evidence both for and against the researcher’s arguments

• If the researcher has discussed the credibility of their findings (e.g. triangulation, respondent validation, more than one analyst)

• If the findings are discussed in relation to the original research question

| **x** |
| --- |
|  |
|  |

**Yes**

**Can’t Tell**

**No**

Section C: Will the results help locally? yes

10- **How valuable is the research?**

HINT: Consider

• If the researcher discusses the contribution the study makes to existing knowledge or understanding (e.g. do they consider the findings in relation to current practice or policy, or relevant research based literature

• If they identify new areas where research is necessary

• If the researchers have discussed whether or how the findings can be transferred to other populations or considered other ways the research may be used

| **x** |
| --- |
|  |
|  |

**Yes**

**Can’t Tell**

**No**

- **Li J, Varnfield M, Jayasena R, Celler B. Home telemonitoring for chronic disease management: Perceptions of users and factors influencing adoption. Health Informatics J. 2021 Jan-Mar;27(1):1460458221997893. doi: 10.1177/1460458221997893. PMID: 33685279**

Section A: Are the results valid? yes

1-Was there a clear statement of the aims of the research?

| x |
| --- |
|  |
|  |

Yes

Can’t Tell

No

Comments: This paper presents an investigation of patients’ and clinicians’ experiences in a care augmenting telemonitoring service, their perceived impact delivered through the service, and clinicians’ perceptions on how the service was introduced in their organizations.

**Limitations:** small convenience sample with more experienced professionals. This can cause bias.

**2-Is a qualitative methodology appropriate?**

| x |
| --- |
|  |
|  |

Yes

HINT: Consider

• If the research seeks to interpret or illuminate the actions and/or subjective experiences of research participants

• Is qualitative research the right methodology for addressing the research goal

Can’t Tell

No

Comments: Clinicians’ experiences and perceptions on the effect of in-home telemonitoring service assessed through questionnaire and semi-structured interviews.

Is it worth continuing? No

**3- Was the research design appropriate to address the aims of the research?**

| x |
| --- |
|  |
|  |

Yes

HINT: Consider

• if the researcher has justified the research design (e.g. have they discussed how they decided which method to use)

Can’t Tell

No

4 **- Was the recruitment strategy appropriate to the aims of the research?**

HINT: Consider

• If the researcher has explained how the participants were selected

• If they explained why the participants they selected were the most appropriate to provide access to the type of knowledge sought by the study

• If there are any discussions around recruitment (e.g. why some people chose not to take part)

|  |
| --- |
|  |
| x |

Yes

Can’t Tell

No

Comments: Convenience non-probabilistic sampling.

5- **Was the data collected in a way that addressed the research issue?**

| x |
| --- |
|  |
|  |

Yes

HINT: Consider

• If the setting for the data collection was justified

• If it is clear how data were collected (e.g. focus group, semi-structured interview etc.)

• If the researcher has justified the methods chosen

• If the researcher has made the methods explicit (e.g. for interview method, is there an indication of how interviews are conducted, or did they use a topic guide)

• If methods were modified during the study. If so, has the researcher explained how and why

• If the form of data is clear (e.g. tape recordings, video material, notes etc.)

• If the researcher has discussed saturation of data

Can’t Tell

No

6- **Has the relationship between researcher and participants been adequately considered?**

HINT: Consider

• If the researcher critically examined their own role, potential bias and influence during (a) formulation of the research questions (b) data collection, including sample recruitment and choice of location

• How the researcher responded to events during the study and whether they considered the implications of any changes in the research design

|  |
| --- |
|  |
| **x** |

**Yes**

**Can’t Tell**

**No**

Comments: The researcher does not talk about bias or ways to deal with them.

7- **Have ethical issues been taken into consideration?**

HINT: Consider

• If there are sufficient details of how the research was explained to participants for the reader to assess whether ethical standards were maintained

• If the researcher has discussed issues raised by the study (e.g. issues around informed consent or confidentiality or how they have handled the effects of the study on the participants during and after the study)

• If approval has been sought from the ethics committee

| **x** |
| --- |
|  |
|  |

**Yes**

**Can’t Tell**

**No**

Comments: Ethics approvals for the trial including the study reported in this paper were granted by the CSIRO CAFHS Human Research Ethics Committee (#13/04) and health authorities at each of the test sites.

8- **Was the data analysis sufficiently rigorous?**

HINT:

• If there is an in-depth description of the analysis process • If thematic analysis is used. If so, is it clear how the categories/themes were derived from the data

• Whether the researcher explains how the data presented were selected from the original sample to demonstrate the analysis process

• If sufficient data are presented to support the findings

• To what extent contradictory data are taken into account • Whether the researcher critically examined their own role, potential bias and influence during analysis and selection of data for presentation

| **x** |
| --- |
|  |
|  |

**Yes**

**Can’t Tell**

**No**

Comments: Although not very detailed.

9**- Is there a clear statement of findings?**

HINT: Consider

• • If the findings are explicit

• If there is adequate discussion of the evidence both for and against the researcher’s arguments

• If the researcher has discussed the credibility of their findings (e.g. triangulation, respondent validation, more than one analyst)

• If the findings are discussed in relation to the original research question

| **x** |
| --- |
|  |
|  |

**Yes**

**Can’t Tell**

**No**

Section C: Will the results help locally? yes

10- **How valuable is the research?**

HINT: Consider

• If the researcher discusses the contribution the study makes to existing knowledge or understanding (e.g. do they consider the findings in relation to current practice or policy, or relevant research based literature

• If they identify new areas where research is necessary

• If the researchers have discussed whether or how the findings can be transferred to other populations or considered other ways the research may be used

| **x** |
| --- |
|  |
|  |

**Yes**

**Can’t Tell**

**No**

- **Alves LFPA, Maia MM, Araújo MFM, Damasceno MMC, Freitas RWJF. Development and validation of a MHEALTH technology for the promotion of self-care for adolescents with diabetes. Cien Saude Colet. 2021 May;26(5):1691-1700. Portuguese, English. doi: 10.1590/1413-81232021265.04602021. PMID: 34076111**

Section A: Are the results valid? yes

1-Was there a clear statement of the aims of the research?

| x |
| --- |
|  |
|  |

Yes

Can’t Tell

No

Comments: To develop and validate by version (1.0) of the Smartphone Usability Questionnaire (SURE), a mobile application (APP) to promote self-care for adolescents with DM1.

**Limitations:** small convenience sample with more experienced professionals. This can cause bias.

**2-Is a qualitative methodology appropriate?**

| x |
| --- |
|  |
|  |

Yes

HINT: Consider

• If the research seeks to interpret or illuminate the actions and/or subjective experiences of research participants

• Is qualitative research the right methodology for addressing the research goal

Can’t Tell

No

Is it worth continuing? No

**3- Was the research design appropriate to address the aims of the research?**

| x |
| --- |
|  |
|  |

Yes

HINT: Consider

• if the researcher has justified the research design (e.g. have they discussed how they decided which method to use)

Can’t Tell

No

4 **- Was the recruitment strategy appropriate to the aims of the research?**

HINT:

• If the researcher has explained how the participants were selected

• If they explained why the participants they selected were the most appropriate to provide access to the type of knowledge sought by the study

• If there are any discussions around recruitment (e.g. why some people chose not to take part)

|  |
| --- |
|  |
| x |

Yes

Can’t Tell

No

Comments: The process of choosing content judges and technicians was carried out by means of a site survey of the Lattes Platform, on the Lattes Curriculum tab, indicating the option “Search Curriculum Lattes”. It wasn't very detailed.

5- **Was the data collected in a way that addressed the research issue?**

| x |
| --- |
|  |
|  |

Yes

HINT: Consider

• If the setting for the data collection was justified

• If it is clear how data were collected (e.g. focus group, semi-structured interview etc.)

• If the researcher has justified the methods chosen

• If the researcher has made the methods explicit (e.g. for interview method, is there an indication of how interviews are conducted, or did they use a topic guide)

• If methods were modified during the study. If so, has the researcher explained how and why

• If the form of data is clear (e.g. tape recordings, video material, notes etc.)

• If the researcher has discussed saturation of data

Can’t Tell

No

6- **Has the relationship between researcher and participants been adequately considered?**

HINT: Consider

• If the researcher critically examined their own role, potential bias and influence during (a) formulation of the research questions (b) data collection, including sample recruitment and choice of location

• How the researcher responded to events during the study and whether they considered the implications of any changes in the research design

|  |
| --- |
|  |
| **x** |

**Yes**

**Can’t Tell**

**No**

Comments: The researcher did not critically examine his own role, potential bias, and influence during (a) formulation of research questions (b) data collection, including sample recruitment and site choice

7- **Have ethical issues been taken into consideration?**

HINT: Consider

• If there are sufficient details of how the research was explained to participants for the reader to assess whether ethical standards were maintained

• If the researcher has discussed issues raised by the study (e.g. issues around informed consent or confidentiality or how they have handled the effects of the study on the participants during and after the study)

• If approval has been sought from the ethics committee

| **x** |
| --- |
|  |
|  |

**Yes**

**Can’t Tell**

**No**

Comments:

The present study followed the precepts ethics of research with human beings. Initially, the research protocol was submitted to Plataforma Brasil and then it was appreciated and approved by the research Ethics Committee in Human Beings (CEP) of the State University from Ceara (UECE).

8- **Was the data analysis sufficiently rigorous?**

HINT: Consider

• If there is an in-depth description of the analysis process • If thematic analysis is used. If so, is it clear how the categories/themes were derived from the data

• Whether the researcher explains how the data presented were selected from the original sample to demonstrate the analysis process

• If sufficient data are presented to support the findings

• To what extent contradictory data are taken into account • Whether the researcher critically examined their own role, potential bias and influence during analysis and selection of data for presentation

|  |
| --- |
|  |
| **x** |

**Yes**

**Can’t Tell**

**No**

Comments:

Content judges responded to the 21 items of the SAM instrument, distributed in six evaluative aspects (1. Content; 2. Requirement of Literacy; 3. Illustrations; 4. Layout and presentation; 5. Stimulation/motivation of learning; 6. Cultural adequacy), indicating: partially adequate, adequate or excellent. In its turn, the technical judges responded to the 31 items of the SURE questionnaire, indicating: 1=strongly disagree; 2=disagree; 3=agree; 4=agree totally. The researcher does not adequately address the methods.

9**- Is there a clear statement of findings?**

HINT: Consider

• If the findings are explicit

• If there is adequate discussion of the evidence both for and against the researcher’s arguments

• If the researcher has discussed the credibility of their findings (e.g. triangulation, respondent validation, more than one analyst)

• If the findings are discussed in relation to the original research question

|  |
| --- |
|  |
| **x** |

**Yes**

**Can’t Tell**

**No**

Comments: Discussion of evidence and presentation of results extremely short and superficial

Section C: Will the results help locally? yes

10- **How valuable is the research?**

HINT: Consider

• If the researcher discusses the contribution the study makes to existing knowledge or understanding (e.g. do they consider the findings in relation to current practice or policy, or relevant research based literature

• If they identify new areas where research is necessary

• If the researchers have discussed whether or how the findings can be transferred to other populations or considered other ways the research may be used

| **x** |
| --- |
|  |
|  |

**Yes**

**Can’t Tell**

**No**

- **Al-Sofiani ME, Alyusuf EY, Alharthi S, Alguwaihes AM, Al-Khalifah R, Alfadda A. Rapid Implementation of a Diabetes Telemedicine Clinic During the Coronavirus Disease 2019 Outbreak: Our Protocol, Experience, and Satisfaction Reports in Saudi Arabia. J Diabetes Sci Technol. 2021 Mar;15(2):329-338. doi: 10.1177/1932296820947094. Epub 2020 Aug 7. PMID: 32762362; PMCID: PMC7925440**

Section A: Are the results valid? yes

1-Was there a clear statement of the aims of the research?

| x |
| --- |
|  |
|  |

Yes

Can’t Tell

No

Comments:

our goal was to design a telemedicine clinic that is simple, practical, and sustainable over a short period of time utilizing tools that were available to us and to our patients at the time.

Healthcare provider’s satisfaction with the diabetes telemedicine Clinic protocol through a satisfaction survey.

**Limitations:** small convenience sample with more experienced professionals. This can cause bias.

**2-Is a qualitative methodology appropriate?**

| x |
| --- |
|  |
|  |

Yes

HINT: Consider

• If the research seeks to interpret or illuminate the actions and/or subjective experiences of research participants

• Is qualitative research the right methodology for addressing the research goal

Can’t Tell

No

Is it worth continuing? No

**3- Was the research design appropriate to address the aims of the research?**

| x |
| --- |
|  |
|  |

Yes

HINT: Consider

• if the researcher has justified the research design (e.g. have they discussed how they decided which method to use)

Can’t Tell

No

4 **- Was the recruitment strategy appropriate to the aims of the research?**

HINT: Consider

• If the researcher has explained how the participants were selected

• If they explained why the participants they selected were the most appropriate to provide access to the type of knowledge sought by the study

• If there are any discussions around recruitment (e.g. why some people chose not to take part)

|  |
| --- |
|  |
| x |

Yes

Can’t Tell

No

Comments: Convenience non-probabilistic sampling.

5- **Was the data collected in a way that addressed the research issue?**

| x |
| --- |
|  |
|  |

Yes

HINT: Consider

• If the setting for the data collection was justified

• If it is clear how data were collected (e.g. focus group, semi-structured interview etc.)

• If the researcher has justified the methods chosen

• If the researcher has made the methods explicit (e.g. for interview method, is there an indication of how interviews are conducted, or did they use a topic guide)

• If methods were modified during the study. If so, has the researcher explained how and why

• If the form of data is clear (e.g. tape recordings, video material, notes etc.)

• If the researcher has discussed saturation of data

Can’t Tell

No

6- **Has the relationship between researcher and participants been adequately considered?**

HINT: Consider

• If the researcher critically examined their own role, potential bias and influence during (a) formulation of the research questions (b) data collection, including sample recruitment and choice of location

• How the researcher responded to events during the study and whether they considered the implications of any changes in the research design

|  |
| --- |
|  |
| **x** |

**Yes**

**Can’t Tell**

**No**

Comments: The researcher did not critically examine his own role, potential bias, and influence during (a) formulation of research questions (b) data collection, including sample recruitment and site choice

7- **Have ethical issues been taken into consideration?**

HINT:

• If there are sufficient details of how the research was explained to participants for the reader to assess whether ethical standards were maintained

• If the researcher has discussed issues raised by the study (e.g. issues around informed consent or confidentiality or how they have handled the effects of the study on the participants during and after the study)

• If approval has been sought from the ethics committee

|  |
| --- |
|  |
| **X** |

**Yes**

**Can’t Tell**

**No**

Comments: It does not make it clear whether it has ethical approval

8- **Was the data analysis sufficiently rigorous?**

HINT: Consider

• If there is an in-depth description of the analysis process • If thematic analysis is used. If so, is it clear how the categories/themes were derived from the data

• Whether the researcher explains how the data presented were selected from the original sample to demonstrate the analysis process

• If sufficient data are presented to support the findings

• To what extent contradictory data are taken into account • Whether the researcher critically examined their own role, potential bias and influence during analysis and selection of data for presentation

|  |
| --- |
|  |
| **x** |

**Yes**

**Can’t Tell**

**No**

Comments:

9**- Is there a clear statement of findings?**

HINT: Consider

• • If the findings are explicit

• If there is adequate discussion of the evidence both for and against the researcher’s arguments

• If the researcher has discussed the credibility of their findings (e.g. triangulation, respondent validation, more than one analyst)

• If the findings are discussed in relation to the original research question

| **x** |
| --- |
|  |
|  |

**Yes**

**Can’t Tell**

**No**

Section C: Will the results help locally? yes

10- **How valuable is the research?**

HINT: Consider

• If the researcher discusses the contribution the study makes to existing knowledge or understanding (e.g. do they consider the findings in relation to current practice or policy, or relevant research based literature

• If they identify new areas where research is necessary

• If the researchers have discussed whether or how the findings can be transferred to other populations or considered other ways the research may be used

| **x** |
| --- |
|  |
|  |

**Yes**

**Can’t Tell**

**No**

- **Marcolino MS, Oliveira JAQ, Cimini CCR, Maia JX, Pinto VSOA, Sá TQV, Amancio K, Coelho L, Ribeiro LB, Cardoso CS, Ribeiro AL. Development and Implementation of a Decision Support System to Improve Control of Hypertension and Diabetes in a Resource-Constrained Area in Brazil: Mixed Methods Study. J Med Internet Res. 2021 Jan 11;23(1):e18872. doi: 10.2196/18872. PMID: 33427686; PMCID: PMC7834943**

Section A: Are the results valid? yes

1-Was there a clear statement of the aims of the research?

| x |
| --- |
|  |
|  |

Yes

Can’t Tell

No

Comments:

The aim was to develop a Clinical decision support system (CDSS) for diabetes and hypertension management in primary care, implement it in a low-income and primarily rural region, and evaluate its usability and healthcare practitioner satisfaction.

Usability and satisfaction of a decision support system to improve health care and control hypertension and diabetes in a resource-constrained area in Brazil through a questionnaire. The questionnaire included 24 items on impressions of feasibility, usability, utility, and satisfaction and presented a global Cronbach α of .93.

**Limitations:** small convenience sample with more experienced professionals. This can cause bias.

**2-Is a qualitative methodology appropriate?**

| x |
| --- |
|  |
|  |

Yes

HINT: Consider

• If the research seeks to interpret or illuminate the actions and/or subjective experiences of research participants

• Is qualitative research the right methodology for addressing the research goal

Can’t Tell

No

Is it worth continuing? No

**3- Was the research design appropriate to address the aims of the research?**

| x |
| --- |
|  |
|  |

Yes

HINT: Consider

• if the researcher has justified the research design (e.g. have they discussed how they decided which method to use)

Can’t Tell

No

Comments: quasi-experimental study.

4 **- Was the recruitment strategy appropriate to the aims of the research?**

HINT: Consider

• If the researcher has explained how the participants were selected

• If they explained why the participants they selected were the most appropriate to provide access to the type of knowledge sought by the study

• If there are any discussions around recruitment (e.g. why some people chose not to take part)

|  |
| --- |
|  |
| x |

Yes

Can’t Tell

No

Comments: Convenience non-probabilistic sampling.

5- **Was the data collected in a way that addressed the research issue?**

| x |
| --- |
|  |
|  |

Yes

HINT: Consider

• If the setting for the data collection was justified

• If it is clear how data were collected (e.g. focus group, semi-structured interview etc.)

• If the researcher has justified the methods chosen

• If the researcher has made the methods explicit (e.g. for interview method, is there an indication of how interviews are conducted, or did they use a topic guide)

• If methods were modified during the study. If so, has the researcher explained how and why

• If the form of data is clear (e.g. tape recordings, video material, notes etc.)

• If the researcher has discussed saturation of data

Can’t Tell

No

6- **Has the relationship between researcher and participants been adequately considered?**

HINT: Consider

• If the researcher critically examined their own role, potential bias and influence during (a) formulation of the research questions (b) data collection, including sample recruitment and choice of location

• How the researcher responded to events during the study and whether they considered the implications of any changes in the research design

|  |
| --- |
|  |
| **x** |

**Yes**

**Can’t Tell**

**No**

7- **Have ethical issues been taken into consideration?**

HINT: Consider

• If there are sufficient details of how the research was explained to participants for the reader to assess whether ethical standards were maintained

• If the researcher has discussed issues raised by the study (e.g. issues around informed consent or confidentiality or how they have handled the effects of the study on the participants during and after the study)

• If approval has been sought from the ethics committee

| **x** |
| --- |
|  |
|  |

**Yes**

**Can’t Tell**

**No**

Comments: Ethical approval was obtained from the Universidade Federal dos Vales do Jequitinhonha e Mucuri Research Ethics Committee (number 65808517.9.0000.5108). Written and informed consent was obtained from all participants.

8- **Was the data analysis sufficiently rigorous?**

HINT: Consider

• If there is an in-depth description of the analysis process • If thematic analysis is used. If so, is it clear how the categories/themes were derived from the data

• Whether the researcher explains how the data presented were selected from the original sample to demonstrate the analysis process

• If sufficient? data are presented to support the findings

• To what extent contradictory data are taken into account • Whether the researcher critically examined their own role, potential bias and influence during analysis and selection of data for presentation

| **x** |
| --- |
|  |
|  |

**Yes**

**Can’t Tell**

**No**

9**- Is there a clear statement of findings?**

HINT: Consider

• • If the findings are explicit

• If there is adequate discussion of the evidence both for and against the researcher’s arguments

• If the researcher has discussed the credibility of their findings (e.g. triangulation, respondent validation, more than one analyst)

• If the findings are discussed in relation to the original research question

| **x** |
| --- |
|  |
|  |

**Yes**

**Can’t Tell**

**No**

Section C: Will the results help locally? yes

10- **How valuable is the research?**

HINT: Consider

• If the researcher discusses the contribution the study makes to existing knowledge or understanding (e.g. do they consider the findings in relation to current practice or policy, or relevant research based literature

• If they identify new areas where research is necessary

• If the researchers have discussed whether or how the findings can be transferred to other populations or considered other ways the research may be used

| **x** |
| --- |
|  |
|  |

**Yes**

**Can’t Tell**

**No**

- **Yee LM, Leziak K, Jackson J, Strohbach A, Saber R, Niznik CM, Simon MA. Patient and Provider Perspectives on a Novel Mobile Health Intervention for Low-Income Pregnant Women With Gestational or Type 2 Diabetes Mellitus. J Diabetes Sci Technol. 2021 Sep;15(5):1121-1133. doi: 10.1177/1932296820937347. Epub 2020 Jul 5. PMID: 32627582; PMCID: PMC8442184**

Section A: Are the results valid? yes

1-Was there a clear statement of the aims of the research?

| x |
| --- |
|  |
|  |

Yes

Can’t Tell

No

Comments:

This prospective, qualitative study was designed to elicit feedback from patients and healthcare providers regarding usability of the SweetMama prototype.

**Limitations:** small convenience sample with more experienced professionals. This can cause bias.

**2-Is a qualitative methodology appropriate?**

| x |
| --- |
|  |
|  |

Yes

HINT: Consider

• If the research seeks to interpret or illuminate the actions and/or subjective experiences of research participants

• Is qualitative research the right methodology for addressing the research goal

Can’t Tell

No

Is it worth continuing? No

**3- Was the research design appropriate to address the aims of the research?**

| x |
| --- |
|  |
|  |

Yes

HINT: Consider

• if the researcher has justified the research design (e.g. have they discussed how they decided which method to use)

Can’t Tell

No

Comments: Qualitative study

4 **- Was the recruitment strategy appropriate to the aims of the research?**

HINT:

• If the researcher has explained how the participants were selected

• If they explained why the participants they selected were the most appropriate to provide access to the type of knowledge sought by the study

• If there are any discussions around recruitment (e.g. why some people chose not to take part)

|  |
| --- |
|  |
| x |

Yes

Can’t Tell

No

Comments: It only addresses patient recruitment criteria.

Any provider with experience treating or educating pregnant women with DM was eligible for participation. Eligible providers included physicians, nurses, medical assistants, and health educators. All providers were over age 18 and English-speaking. Provider participants were recruited by conducting a review of the institution’s clinical personnel records. Provider focus groups were intentionally composed of providers of different types. All providers used their own smartphones for participation.

a weakness in this study is its inclusion of participants from a single medical center, which may limit generalizability. Importantly, this study was limited to English-speaking women, and thus the majority of the patient population was non-Hispanic black.

Convenience non-probabilistic sampling.

5- **Was the data collected in a way that addressed the research issue?**

| x |
| --- |
|  |
|  |

Yes

HINT: Consider

• If the setting for the data collection was justified

• If it is clear how data were collected (e.g. focus group, semi-structured interview etc.)

• If the researcher has justified the methods chosen

• If the researcher has made the methods explicit (e.g. for interview method, is there an indication of how interviews are conducted, or did they use a topic guide)

• If methods were modified during the study. If so, has the researcher explained how and why

• If the form of data is clear (e.g. tape recordings, video material, notes etc.)

• If the researcher has discussed saturation of data

Can’t Tell

No

6- **Has the relationship between researcher and participants been adequately considered?**

HINT: Consider

• If the researcher critically examined their own role, potential bias and influence during (a) formulation of the research questions (b) data collection, including sample recruitment and choice of location

• How the researcher responded to events during the study and whether they considered the implications of any changes in the research design

|  |
| --- |
|  |
| **x** |

**Yes**

**Can’t Tell**

**No**

7- **Have ethical issues been taken into consideration?**

HINT: Consider

• If there are sufficient details of how the research was explained to participants for the reader to assess whether ethical standards were maintained

• If the researcher has discussed issues raised by the study (e.g. issues around informed consent or confidentiality or how they have handled the effects of the study on the participants during and after the study)

• If approval has been sought from the ethics committee

| **x** |
| --- |
|  |
|  |

**Yes**

**Can’t Tell**

**No**

Comments: This study was approved by the Northwestern University Institutional Review Board.

8- **Was the data analysis sufficiently rigorous?**

HINT: Consider

• If there is an in-depth description of the analysis process • If thematic analysis is used. If so, is it clear how the categories/themes were derived from the data

• Whether the researcher explains how the data presented were selected from the original sample to demonstrate the analysis process

• If sufficient data are presented to support the findings

• To what extent contradictory data are taken into account • Whether the researcher critically examined their own role, potential bias and influence during analysis and selection of data for presentation

| **x** |
| --- |
|  |
|  |

**Yes**

**Can’t Tell**

**No**

9**- Is there a clear statement of findings?**

HINT: Consider

• • If the findings are explicit

• If there is adequate discussion of the evidence both for and against the researcher’s arguments

• If the researcher has discussed the credibility of their findings (e.g. triangulation, respondent validation, more than one analyst)

• If the findings are discussed in relation to the original research question

| **x** |
| --- |
|  |
|  |

**Yes**

**Can’t Tell**

**No**

Section C: Will the results help locally? yes

10- **How valuable is the research?**

HINT: Consider

• If the researcher discusses the contribution the study makes to existing knowledge or understanding (e.g. do they consider the findings in relation to current practice or policy, or relevant research based literature

• If they identify new areas where research is necessary

• If the researchers have discussed whether or how the findings can be transferred to other populations or considered other ways the research may be used

| **x** |
| --- |
|  |
|  |

**Yes**

**Can’t Tell**

**No**

- **Salari R, R Niakan Kalhori S, GhaziSaeedi M, Jeddi M, Nazari M, Fatehi F. Mobile-Based and Cloud-Based System for Self-management of People With Type 2 Diabetes: Development and Usability Evaluation. J Med Internet Res. 2021 Jun 2;23(6):e18167. doi: 10.2196/18167. PMID: 34076579; PMCID: PMC8209530.**

Section A: Are the results valid? yes

1-Was there a clear statement of the aims of the research?

| x |
| --- |
|  |
|  |

Yes

Can’t Tell

No

Comments: Usability of a cloud-based and mobile-based diabetes self-management app, using the User Experience Questionnaire (UEQ).

**Limitations:** small convenience sample with more experienced professionals. This can cause bias.

**2-Is a qualitative methodology appropriate?**

| x |
| --- |
|  |
|  |

Yes

HINT: Consider

• If the research seeks to interpret or illuminate the actions and/or subjective experiences of research participants

• Is qualitative research the right methodology for addressing the research goal

Can’t Tell

No

Is it worth continuing? No

**3- Was the research design appropriate to address the aims of the research?**

| x |
| --- |
|  |
|  |

Yes

HINT: Consider

• if the researcher has justified the research design (e.g. have they discussed how they decided which method to use)

Can’t Tell

No

Comments: Qualitative study.

4 **- Was the recruitment strategy appropriate to the aims of the research?**

HINT: Consider

• If the researcher has explained how the participants were selected

• If they explained why the participants they selected were the most appropriate to provide access to the type of knowledge sought by the study

• If there are any discussions around recruitment (e.g. why some people chose not to take part)

|  |
| --- |
|  |
| x |

Yes

Can’t Tell

No

5- **Was the data collected in a way that addressed the research issue?**

| x |
| --- |
|  |
|  |

Yes

HINT: Consider

• If the setting for the data collection was justified

• If it is clear how data were collected (e.g. focus group, semi-structured interview etc.)

• If the researcher has justified the methods chosen

• If the researcher has made the methods explicit (e.g. for interview method, is there an indication of how interviews are conducted, or did they use a topic guide)

• If methods were modified during the study. If so, has the researcher explained how and why

• If the form of data is clear (e.g. tape recordings, video material, notes etc.)

• If the researcher has discussed saturation of data

Can’t Tell

No

6- **Has the relationship between researcher and participants been adequately considered?**

HINT: Consider

• If the researcher critically examined their own role, potential bias and influence during (a) formulation of the research questions (b) data collection, including sample recruitment and choice of location

• How the researcher responded to events during the study and whether they considered the implications of any changes in the research design

|  |
| --- |
|  |
| **x** |

**Yes**

**Can’t Tell**

**No**

7- **Have ethical issues been taken into consideration?**

HINT: Consider

• If there are sufficient details of how the research was explained to participants for the reader to assess whether ethical standards were maintained

• If the researcher has discussed issues raised by the study (e.g. issues around informed consent or confidentiality or how they have handled the effects of the study on the participants during and after the study)

• If approval has been sought from the ethics committee

|  |
| --- |
|  |
| **x** |

**Yes**

**Can’t Tell**

**No**

8- **Was the data analysis sufficiently rigorous?**

HINT: Consider

• If there is an in-depth description of the analysis process • If thematic analysis is used. If so, is it clear how the categories/themes were derived from the data

• Whether the researcher explains how the data presented were selected from the original sample to demonstrate the analysis process

• If sufficient data are presented to support the findings

• To what extent contradictory data are taken into account • Whether the researcher critically examined their own role, potential bias and influence during analysis and selection of data for presentation

| **x** |
| --- |
|  |
|  |

**Yes**

**Can’t Tell**

**No**

9**- Is there a clear statement of findings?**

HINT: Consider

• • If the findings are explicit

• If there is adequate discussion of the evidence both for and against the researcher’s arguments

• If the researcher has discussed the credibility of their findings (e.g. triangulation, respondent validation, more than one analyst)

• If the findings are discussed in relation to the original research question

| **x** |
| --- |
|  |
|  |

**Yes**

**Can’t Tell**

**No**

Section C: Will the results help locally? yes

10- **How valuable is the research?**

HINT: Consider

• If the researcher discusses the contribution the study makes to existing knowledge or understanding (e.g. do they consider the findings in relation to current practice or policy, or relevant research based literature

• If they identify new areas where research is necessary

• If the researchers have discussed whether or how the findings can be transferred to other populations or considered other ways the research may be used

| **x** |
| --- |
|  |
|  |

**Yes**

**Can’t Tell**

**No**
