# Supplementary material for: Usability of Telehealth Systems for Noncommunicable Diseases in Primary Care From the COVID-19 Pandemic Onward: Systematic Review
Source: J Med Internet Res. 2023 Mar 16;25:e44209. doi: 10.2196/44209 (PMC10022651; doi:10.2196/44209)
Supplement: Multimedia Appendix 3 [file jmir_v25i1e44209_app3.docx]

**Multimedia Appendix 3.** Excluded studies and reasons for exclusion.

| **Article, scientific journal, year** | **Reason for exclusion** |
| --- | --- |
| 1. Evaluating the effectiveness and utility of a novel culturally adapted telemonitoring system in improving the glycaemic control of Asians with type-2 diabetes mellitus: a mixed method study protocol. Trials. 2021 | Different outcome than desired |
| 1. Health technology assessment for digital technologies that manage chronic disease: a systematic review. Int J Technol Assess Health Care. 2021 | Different outcome than desired |
| 1. Effectiveness of telemedicine-guided home blood pressure compared to 24 h-ambulatory blood pressure monitoring in patients with and without chronic kidney disease. Arterial Hypertension 2020 | Different outcome than desired |
| 1. TELEmedicine for EPIlepsy Care (TELE-EPIC): protocol of a randomised, open controlled non-inferiority clinical trial. BMJ Open. 2021 | Different outcome than desired |
| 1. Effectiveness of Mobile App-Assisted Self-Care Interventions for Improving Patient Outcomes in Type 2 Diabetes and/or Hypertension: Systematic Review and Meta-Analysis of Randomized Controlled Trials. JMIR Mhealth Uhealth 2020 | Different outcome than desired |
| 1. Biobehavioural Physiotherapy through Telerehabilitation during the SARS-CoV-2 Pandemic in a Patient with Post-polio Syndrome and Low Back Pain: A Case Report. Phys Ther Res. 2021 | Different outcome than desired |
| 1. Mobile phone messaging telemedicine for facilitating self-management of long-term illnesses. Cochrane Database Syst Rev. 2012 | Different outcome than desired |
| 1. Building rapport and earning the surgical patient's trust in the era of social distancing: teaching patient-centered communication during video conference encounters to medical students. J Surg Educ. 2021 | Different outcome than desired |
| 1. How Does Telemedicine Compare to Conventional Follow-Up After General Surgery? Journal of Clinical Outcomes Management. 2020 | Different outcome than desired |
| 1. Mobile Apps for Health Behavior Change: Protocol for a Systematic Review. JMIR Res Protoc. 2020 | Different design than desired |
| 1. Protocol: Codesign and implementation of an equity-promoting national health literacy programme for people living with inflammatory bowel disease (IBD): a protocol for the application of the Optimising Health Literacy and Access (Ophelia) process. [BMJ Open.](https://www.ncbi.nlm.nih.gov/pmc/articles/PMC8395357/) 2021 | Different design than desired |
| 1. Impact of Telehealth Interventions on Medication Adherence for Patients with Type 2 Diabetes, Hypertension, and/or Dyslipidemia: A Systematic Review. Ann Pharmacother. 2021 | Different design than desired |
| 1. Clinical Improvements by Telemedicine Interventions Managing Type 1 and Type 2 Diabetes: Systematic Meta-review. [J Med Internet Res.](https://www.ncbi.nlm.nih.gov/pmc/articles/PMC7935656/) 2021 | Different design than desired |
| 1. Reducing weight and BMI following gestational diabetes: a systematic review and meta-analysis of digital and telemedicine interventions. BMJ Open Diabetes Res Care. 2021 | Different design than desired |
| 1. Detecting and Treating Psychosocial and Lifestyle-Related Difficulties in Chronic Disease: Development and Treatment Protocol of the E-GOAL eHealth Care Pathway. Int J Environ Res Public Health. 2021 | Different outcome than desired |
| 1. Effectiveness of Disease-Specific mHealth Apps in Patients with Diabetes Mellitus: Scoping Review. Mhealth Uhealth. 2021 | Different outcome than desired |
| 1. The Patient-centered Medical Home as an Intervention Strategy for Diabetes Mellitus: A Systematic Review of the Literature. Curr Diabetes Rev. 2021 | Different design than desired |
| 1. Utilizarea soluțiilor digitale în autogestionarea bolilor cardiovasculare și a diabetului zaharat. In: Sănătate Publică, Economie şi Management în Medicină. 2020 | Different outcome than desired |
| 1. Evolution of mHealth eco-system: a step towards personalized medicine. (eds) International Conference on Innovative Computing and Communications. Advances in Intelligent Systems and Computing. 2020 | Different publication type than desired |
| 1. 1980-2019 年糖尿病经济学评价文献计量分析. Disease Surveillance. 2022 | Different language than desired |
| 1. Technology and Adolescent Health: In Schools and Beyond. 2020 | Different publication type than desired |
| 1. Risk Factors for Loss to Follow-Up in the Lower Extremity Limb Salvage Population. Plastic and Reconstructive Surgery. 2021 | Different outcome than desired |
| 1. The Novel Coronavirus Disease (COVID-19) and Its Impact on Cardiovascular Disease. Cardiol Ver. 2020 | Different outcome than desired |
| 1. The importance of knowing and listening to all those involved in the design and use of nutrition mobile apps. Getting to know the Great GApp. Nutr Hosp. 2021 | Different outcome than desired |
| 1. Telehealth and Women’s Perinatal Mental Health. Women's Mental Health. 2020 | Different population than desired |
| 1. The effect of covid-19 pandemic on sarcopenia, quality of life and pain: A one-year follow-up study. Turk Geriatri Dergisi. 2021 | Different population than desired |
| 1. Blended learning compared to traditional learning in medical education: systematic review and meta-analysis. J Med Internet Res 2020 | Different design than desired |
| 1. Environmental Data as Еxposome and Оpportunity of Combining with Cloud-Based Personal Health Records. In: The 14-th conference on Information Systems and Grid Technologies. 2021 | Different publication type than desired |
| 1. What is the Prevalence of Low Health Literacy in European Union Member States? A Systematic Review and Meta-analysis. Journal of General Internal Medicine. 2021 | Different population than desired |
| 1. Tele-neuropsychological assessment tools in Italy: a systematic review on psychometric properties and usability. Neurological Sciences. 2022 | Different design than desired |
| 1. Lipid Management in Patients Presenting With Acute Coronary Syndromes: A Review. J Am Heart Assoc. 2020 | Different population than desired |
| 1. Designing a Health Coach-Augmented mHealth System for the Secondary Prevention of Coronary Heart Disease Among Women. IEEE Transactions on Engineering Management. 2020 | Different population than desired |
| 1. O nouă metodă de determinare și transmitere la distanță a valorilor tensiunii arteriale utilizând sisteme cuffless (Pulse Transit Time). Revista stiintifica a Universatii de Stat din Moldova, 2021 | Different language than desired |
| 1. Deep learning in patient management and clinical decision making. In book: Deep Learning for Personalized Healthcare Services. 2021 | Different publication type than desired |
| 1. Medication adherence changes in Blacks with diabetes: a mixed methods study. American Journal of Health Behavior. 2020 | Different outcome than desired |
| 1. Pulmonary Rehabilitation in COVID-19: The Contribution of other Chronic Lung Syndromes for the Intervention of a Novel Disease. Current Respiratory Medicine Reviews. 2020 | Different population than desired |
| 1. A Survey on the Management of Children with Asthma in Primary Care Setting in Italy. Pediatr Allergy Immunol Pulmonol. 2021 | Different population than desired |
| 1. Information Visualisation Practices for Improving Patient Readability of Blood Pressure, Health Data, and Health Literacy. Thesis. 2020 | Different publication type than desired |
| 1. A review of implementation frameworks to operationalize health technology assessment recommendations for medical technologies in the Singapore setting. Int J Technol Assess Health Care. 2021 | Different design than desired |
| 1. Evaluation, Acceptance, and Qualification of Digital Measures: From Proof of Concept to Endpoint. Digit Biomark. 2021 | Different outcome than desired |
| 1. Chronic Wound Assessment and Treatment System (CWATS). IEEE. 2019 | Different outcome than desired |
| 1. Development and Validation of a Deep Learning Model for Earlier Detection of Cognitive Decline from Clinical Notes in Electronic Health Records. *JAMA* Netw Open*.*2021 | Different outcome than desired |
| 1. Efficacy and acceptability of My Care Hub mobile app to support self-management in Australians with type 1 or type 2 diabetes. Int J Environ Res Public Health. 2020 | Different outcome than desired |
| 1. Referral Patterns of Central Retinal Artery Occlusion to an Academic Center Affiliated with a Stroke Center. J Neuroophthalmol. 2021 | Different outcome than desired |
| 1. Converting and expanding mobile support tools for Tuberculosis treatment support: Design recommendations from domain and design experts. J Biomed Inform X. 2020 | Different population than desired |
| 1. Health-Focused Publicâ¬"Private Partnerships in the Urban Context: Proceedings of a Workshop. Book. 2020 | Different publication type than desired |
| 1. Credentialing and privileging for clinical pharmacists. J Am Coll Clin Pharm.2020 | Different outcome than desired |
| 1. Kronik Hastalıkların Yönetiminde Kronik Bakım Modeline Temellendirilmiş Girişimlerin Etkinliği: Geleneksel Derleme. NURSES.2020 | Different language than desired |
| 1. Technology Based Business Development | Different publication type than desired |
| 1. Creating Healthy Organizations, Revised and Expanded Edition: Taking Action to Improve Employee Well-Being. Book | Different publication type than desired |
| 1. Technology and Mental Health. 2022 | Different publication type than desired |
| 1. DDSEP | Different publication type than desired |
| 1. A self-management application “pain-housekeeper” to control cancer pain: a randomized controlled trial study protocol. Frontiers of nursing. 2020 | Different outcome than desired |
| 1. Cost benefits and mechanisms of implementing an AI triage solution in a primary healthcare center–Case Klinik Access at Myyrmäki health center. Thesis. 2021 | Different publication type than desired |
| 1. Do Women in Nepal Like Playing a Mobile Game? MANTRA: A Mobile Gamified App for Improving Healthcare Seeking Behavior in Rural Nepal. Front Public Health. 2021 | Different outcome than desired |
| 1. The Public Health Turn in Reproductive Rights. Washington and Lee Law Review. 2021 | Different population than desired |
| 1. Authors and Abstracts | Different design than desired |
| 1. Internet of Everything | Different design than desired |
| 1. Patient observation skills in critical care nursing: A Theoretical construction and evaluation. Book. 2021 | Different publication type than desired |
| 1. Paediatric Palliative Care National Action Plan Project: Background Literature Review. PalliativeCare | Different design than desired |
| 1. Co-Production Performance Evaluation in Healthcare. A Systematic Review of Methods, Tools and Metrics. Int J Environ Res Public Health. 2021 | Different population than desired |
| 1. Improving patient disposition to rapid access clinic models of care following emergency department discharge. Emergency Medicine Australasia. Poster Presentations. 2020 | Different publication type than desired |
| 1. Paramedic Attitudes and Perceptions About Continuing Professional Development in Australasia. Conference. 2020 | Different publication type than desired |
| 1. Supporting information for the rapid evidence synthesis (IM stage 1). | Different publication type than desired |
| 1. Stepped care treatment delivery for depression: a systematic review and meta-analysis. Psychol Med. 2015 | Different design than desired |
| 1. Alberta Rating Index for Apps (ARIA): An Index to Rate the Quality of Mobile Health Applications. Can J Occup Ther. 2022 | Different outcome than desired |
| 1. Pediatric Neurology: Clinical Assessment and Management. Book | Different publication type than desired |
| 1. Oral Epidemiology: A Textbook on Oral Health Conditions, Research Topics, and Methods. Book | Different publication type than desired |
| 1. Development of an interactive tool to support the evaluation of clinic-based health information systems. Thesis. 2021 | Different publication type than desired |
| 1. Precision Health and Genomics. | Different publication type than desired |
| 1. Early Health Technology Assessment of E-health. Thesis. 2021 | Different publication type than desired |
| 1. AACP REPORT School Posters Presented at the 121st Virtual Annual Meeting of the American Association of Colleges of Pharmacy. 2020. | Different publication type than desired |
| 1. Workplace-based interventions to promote healthy lifestyles in the NHS workforce: a rapid scoping and evidence map. NIHR Journals Library. 2020 | Different design than desired |
| 1. 虚血性心疾患患者に対する面談を含むモバイルアプリケー ションを用いた介入効果に関する系統的レビュ | Different language than desired |
| 1. Opioid Use Disorder and Education and MAT Waiver Rapid Review Search Strategy. Temple university. 2021 | Different question than desired |
| 1. SUPNPXD ID>¡ XI]¡ JEDVX]¡ DV JO XID/) 6 [PUMEPUADs> U> SJC VAPSJOH> OC DZJCDOAD N> S | Different publication type than desired |
| 1. ROMSKÝ SENIOR VE VZTAHU K RODINĚ A SPOLEČNOSTI | Different publication type than desired |
| 1. CGS 2020 Book of Abstracts. | Different design than desired |
| 1. The Health Services Executive (HSE): Tools for Leading Long-term Care and Senior Living Organizations. Book. 2020 | Different publication type than desired |
| 1. Φαρμακολογικό προφίλ των τρεχουσών φαρμακευτικών προσεγγίσεων για τη θεραπεία του COVID-19. | Different language than desired |
| 1. Perturbation de la rythmicité circadienne: impact sur la fonction reproductive de souris femelles. Thesis. 2021 | Different publication type than desired |
| 1. Foundations of mental health promotion. Book | Different publication type than desired |
| 1. Proceedings from the 13th Annual Conference on the Science of Dissemination and Implementation. Conference. 2013 | Different publication type than desired |
| 1. Measuring, predicting, and tracking change in psychotherapy. Chapter. 2021 | Different publication type than desired |
| 1. A Uniform Intelligent Prioritisation for Solving Diverse and Big Data Generated from Multiple Chronic Diseases Patients Based on Hybrid Decision-Making and Voting Method. IEEE Access. 2020 | Different outcome than desired |
| 1. Pattern Recognition Reveals Characteristic Postprandial Glucose Changes: Non-Individualized Meal Detection in Diabetes Mellitus Type 1. IEEE J Biomed Health Inform 2020 | Different question than desired |
| 1. Remote Physical Frailty Monitoring– The Application of Deep Learning-Based Image Processing in Tele-Health. IEEE Access. 2020 | Different question than desired |
| 1. Deep Learning for Diabetes: A Systematic Review. IEEE Journal of Biomedical and Health Informatics. 2021 | Different design than desired |
| 1. The Digital/Virtual Diabetes Clinic: The Future Is Now-Recommendations from an International Panel on Diabetes Digital Technologies Introduction. Diabetes Technol Ther. 2021 | Different question than desired |
| 1. Human Activity Recognition with Device-Free Sensors for Well-Being Assessment in Smart Homes. IEEE Instrumentation & Measurement Magazine. 2021 | Different question than desired |
| 1. Design and usability evaluation of COOK, an assistive technology for meal preparation for persons with severe TBI. Disabil Rehabil Assist Technol. 2021 | Different population than desired |
| 1. Development and evaluation of a mobile patient application to enhance medical-dental integration for the treatment of periodontitis and diabetes. Int J Med Inform. 2021 | Different question than desired |
| 1. An Evaluation of Two Capillary Sample Collection Kits for Laboratory Measurement of HbA1c. Diabetes Technol Ther. 2021 | Different question than desired |
| 1. Evaluating the usability and safety of the semaglutide single-dose pen-injectors through summative (human factors) usability testing. J Diabetes Investig. 2021 | Different question than desired |
| 1. Optimizing Engagement in an mHealth Intervention for Diabetes Support During Pregnancy: The Role of Baseline Patient Health and Behavioral Characteristics. J Diabetes Sci Technol. 2021 | Different question than desired |
| 1. Formative and Validation Human Factors studies of a new disposable autoinjector for subcutaneous delivery of chronic disease therapies. Expert Opin Drug Deliv. 2021 | Different question than desired |
| 1. Semaglutide single-dose pen-injector: Post hoc analysis of summative usability testing for weight management. | Different population than desired |
| 1. Home and Online Management and Evaluation of Blood Pressure (HOME BP) using a digital intervention in poorly controlled hypertension: randomised controlled trial. BMJ. 2021 | Different question than desired |
| 1. COVID-19 and diabetes; Possible role of polymorphism and rise of telemedicine. Prim Care Diabetes. 2021 | Different question than desired |
| 1. Management of patients with diabetes and obesity in the COVID-19 era: Experiences and learnings from South and East Europe, the Middle East, and Africa. Diabetes Res Clin Pract. 2021 | Different question than desired |
| 1. Practicing With Uncertainty: Kidney Transplantation During the COVID-19 Pandemic. Am J Kidney Dis, 2021 | Different outcome than desired |
| 1. The Lancet Commission on diabetes: using data to transform diabetes care and patient lives. Lancet. 2021 | Different question than desired |
| 1. Specialty COPD care during COVID-19: patient and clinician perspectives on remote delivery. BMJ Open Respir Res. 2021 | Different population than desired |
| 1. The Digital/Virtual Diabetes Clinic: The Future Is Now-Recommendations from an International Panel on Diabetes Digital Technologies Introduction. Diabetes Technol Ther. 2021 | Different design than desired |
| 1. Hypertension delays viral clearance and exacerbates airway hyperinflammation in patients with COVID-19. Nat Biotechnol. 2021 | Different outcome than desired |
| 1. The Associations of Aspirin, Statins, and Metformin with Lung Cancer Risk and Related Mortality: A Time-Dependent Analysis of Population-Based Nationally Representative Data. J Thorac Oncol. 2021 | Different outcome than desired |
| 1. Memory-like HCV-specific CD8(+) T cells retain a molecular scar after cure of chronic HCV infection. Nat Immunol. 2021 | Different population than desired |
| 1. Telehealth in type 1 diabetes. Curr Opin Endocrinol Diabetes Obes. 2021 | Different outcome than desired |
| 1. Digital Health Approaches for the Assessment and Optimisation of Hypertension Care Provision. Can J Cardiol. 2021 | Different outcome than desired |
| 1. Integrated Digital Patient Education at the Bedside for Patients with Chronic Conditions: Observational Study. JMIR Mhealth Uhealth. 2020 | Different outcome than desired |
| 1. Telemedicine, Patient Satisfaction, and Chronic Rhinosinusitis Care in the Era of COVID-19. Am J Rhinol Allergy. 2021 | Different population than desired |
| 1. Chronic viral hepatitis C micro-elimination program using telemedicine. The Mexican experience. Rev Esp Enferm Dig. 2021 | Different population than desired |
| 1. e-Health Interventions for Community-Dwelling Type 2 Diabetes: A Scoping Review. Telemed J E Health. 2021 | Different population than desired |
| 1. Perceptions of Practitioners on Telehealth and App Use for Smoking Cessation and COPD Care-An Exploratory Study. Medicina (Kaunas). 2020 | Different population than desired |
| 1. Health Care Policy and Regulatory Challenges for Adoption of Telemedicine in Kidney Transplantation. American Journal of Kidney Diseases, 2021 | Different population than desired |
| 1. Assessment of Psychological Distress in Adults with Type 2 Diabetes Mellitus Through Technologies: Literature Review. J Med Internet Res. 2021 | Different outcome than desired |
| 1. Adaptation of a mobile phone health survey for risk factors for noncommunicable diseases in Colombia: a qualitative study. Glob Health Action. 2020 | Different outcome than desired |
| 1. Digital health and management of chronic disease: A multimodal technologies typology. Int J Health Plann Manage. 2021 | Different outcome than desired |
| 1. Digital Health Tools for Managing Noncommunicable Diseases During and After the COVID-19 Pandemic: Perspectives of Patients and Caregivers. [J Med Internet Res](https://www.ncbi.nlm.nih.gov/pmc/journals/224/). 2021 | Different outcome than desired |
| 1. Integrating family and friend support, information technology, and diabetes education in community-centric diabetes self-management. J Am Med Inform Assoc. 2021 | Different outcome than desired |
| 1. Prevalence and Predictors of Health-Related Internet and Digital Device Use in a Sample of South Asian Adults in Edmonton, Alberta, Canada: Results From a 2014 Community-Based Survey. JMIR Public Health Surveill. 2021 | Different outcome than desired |
| 1. Implementing Mobile Health-Enabled Integrated Care for Complex Chronic Patients: Intervention Effectiveness and Cost-Effectiveness Study. JMIR Mhealth Uhealth. 2021 | Different outcome than desired |
| 1. Telerehabilitation for Geriatrics. Phys Med Rehabil Clin N Am. 2021 | Different outcome than desired |
| 1. Coronavirus disease-2019: implications for the gastroenterologist. Curr Opin Gastroenterol. 2021 | Different outcome than desired |
| 1. An International, Mixed-Methods Study of the Perceived Intrusiveness of Remote Digital Diabetes Monitoring. Mayo Clin Proc. 2021 | Different outcome than desired |
| 1. Mobile Health (mHealth) Technology: Assessment of Availability, Acceptability, and Use in CKD. Am J Kidney Dis. 2021 | Different population than desired |
| 1. Mobile Health App with social media to Support Self-Management for Patients with Chronic Kidney Disease: Prospective Randomized Controlled Study. J Med Internet Res. 2020 | Different population than desired |
| 1. Telerehabilitation for chronic respiratory disease. [Cochrane Database Syst Rev.](https://www.ncbi.nlm.nih.gov/pmc/articles/PMC8095032/) 2021 | Different outcome than desired |
| 1. Systematic Evaluation of Canadian Diabetes Smartphone Applications for People with Type 1, Type 2 and Gestational Diabetes. Can J Diabetes. 2021 | Different outcome than desired |
| 1. Effect of Telemedicine on Quality of Care in Patients with Coexisting Hypertension and Diabetes: A Systematic Review and Meta-Analysis. Telemed J E Health. 2021 | Different outcome than desired |
| 1. Assessment of chronic disease management mode (CDMM) on participants with primary hypertension. Trop Med Int Health. 2021 | Different outcome than desired |
| 1. Criteria for Assessing and Recommending Digital Diabetes Tools: A Delphi Study. Stud Health Technol Inform. 2021 | Different outcome than desired |
| 1. Using telehealth to assess depression and suicide ideation and provide mental health interventions to groups of chronically ill adolescents and young adults. Res Nurs Health. 2021 | Different outcome than desired |
| 1. Chronic disease management in heart failure: focus on telemedicine and remote monitoring. Rev Cardiovasc Med. 2021 | Different outcome than desired |
| 1. The application of telehealth to remote and rural Australians with chronic neurological conditions. Intern Med J. 2021 | Different outcome than desired |
| 1. Are People with Chronic Diseases Satisfied with the Online Health Information Related to COVID-19 During the Pandemic? J Nurs Scholarsh. 2021 | Different outcome than desired |
| 1. Deep Learning for Diabetes: A Systematic Review. IEEE J Biomed Health Inform. 2021 | Different design than desired |
| 1. Workshops as Tools for Developing Collaborative Practice across Professional Social Worlds in Telemonitoring. Int J Environ Res Public Health. 2020 | Different outcome than desired |
| 1. A Telehealth-supported, Integrated care with CHWs, and MEdication-access (TIME) Program for Diabetes Improves HbA1c: a Randomized Clinical Trial. J Gen Intern Med. 2021 | Different outcome than desired |
| 1. International feasibility study for the Women's Wellness with Type 2 Diabetes Programme (WWDP): An eHealth enabled 12-week intervention programme for midlife women with type 2 diabetes. Diabetes Res Clin Pract. 2021 | Different outcome than desired |
| 1. Video-Enhanced Care Management for Medically Complex Older Adults with Cognitive Impairment. J Am Geriatr Soc. 2021 | Different population than desired |
| 1. Supporting patients to be involved in decisions about their health and care: Development of a best practice health literacy App for Australian adults living with chronic kidney disease. Health Promot J Austral. 2021 | Different population than desired |
| 1. Telemedicine for Disparity Patients with Diabetes: The Feasibility of Utilizing Telehealth in the Management of Uncontrolled Type 2 Diabetes in Black and Hispanic Disparity Patients; A Pilot Study. J Diabetes Sci Technol. 2021 | Different outcome than desired |
| 1. Impact of COVID-19 on Health Economics and Technology of Diabetes Care: Use Cases of Real-Time Continuous Glucose Monitoring to Transform Health Care During a Global Pandemic. Diabetes technology & therapeutics. 2021 | Different outcome than desired |
| 1. Experiences and Beliefs of Low-Income Patients with Hypertension in Louisiana and Mississippi During the COVID-19 Pandemic. J Am Heart Assoc. 2021 2. Checklist for patients with type 2 diabetes mellitus for remote consultation. Aten Primaria. 2021 | Different outcome than desired |
| 1. Technology-enabled remote management of diabetes foot disease and potential for reduction in associated health costs: a pilot study. Journal of Foot and Ankle Research. 2021 | Different outcome than desired |
| 1. Mobile Diabetes Telemedicine Clinics for Aboriginal First Nation People with Reported Diabetes in British Columbia. Can J Diabetes. 2021 | Different outcome than desired |
| 1. Evaluation and management of COVID-19-related severity in people with type 2 diabetes. BMJ Open Diabetes Res Care. 2021 | Different outcome than desired |
| 1. Impact of a diabetes remote monitoring program on medication adherence. J Manag Care Spec Pharm. 2021 | Different outcome than desired |
| 1. Improved blood pressure control via a novel chronic disease management model of care in sub-Saharan Africa: Real-world program implementation results.  [J Clin Hypertens (Greenwich)](https://www.ncbi.nlm.nih.gov/pmc/journals/4028/). 2021 | Different outcome than desired |
| 1. Casting a Health Equity Lens on Endocrinology and Diabetes. J Clin Endocrinol Metab. 2021 | Different outcome than desired |
| 1. Efficacy of telemedicine for persons with type 1 diabetes during Covid19 lockdown. Nutr Diabetes. 2021 | Different outcome than desired |
| 1. Digital interventions self-management education for type 1 and 2 diabetes: A systematic review and meta-analysis. Computer Methods and Programs in Biomedicine. 2021 | Different outcome than desired |
| 1. Mobile Health in Chronic Disease Management and Patient Empowerment: Exploratory Qualitative Investigation into Patient-Physician Consultations. J Med Internet Res. 2021 | Different population than desired |
| 1. Conversational Agents as Mediating Social Actors in Chronic Disease Management Involving Health Care Professionals, Patients, and Family Members: Multisite Single-Arm Feasibility Study. J Med Internet Res. 2021 | Different outcome than desired |
| 1. Value-based approach to blood pressure telemonitoring and remote counseling in hypertensive patients. Blood Press. 2021 | Different outcome than desired |
| 1. Remote Outpatient Management During COVID-19 Lockdown: Patient-Derived Quality Assessment. Qual Manag Health Care. 2021 | Different outcome than desired |
| 1. Digital health interventions for the management of mental health in people with chronic diseases: a rapid review. BMJ Open. 2021 | Different outcome than desired |
| 1. Role of new digital technologies and telemedicine in pulmonary rehabilitation: Smart devices in the treatment of chronic respiratory diseases. Wien Klin Wochenschr. 2021 | Different outcome than desired |
| 1. Shaping Workflows in Digital and Remote Diabetes Care During the COVID-19 Pandemic via Service Design: Prospective, Longitudinal, Open-label Feasibility Trial. JMIR Mhealth Uhealth. 2021 | Different outcome than desired |
| 1. Digital Coaching Strategies to Facilitate Behavioral Change in Type 2 Diabetes: A Systematic Review, The Journal of Clinical Endocrinology & Metabolism, 2021 | Different design than desired |
| 1. Does telerehabilitation result in inferior clinical outcomes compared with in-person care for the management of chronic musculoskeletal spinal conditions in the tertiary hospital setting? A non-randomised pilot clinical trial. J Telemed Telecare. 2021 | Different outcome than desired |
| 1. Telenephrology and on-site nephrology: Comparable adequate dialysis care to patients living in remote Pacific Islands. J Telemed Telecare. 2021 | Different outcome than desired |
| 1. Assessing telehealth interventions for physical activity and sedentary behavior self-management in adults with type 2 diabetes mellitus: An integrative review. | Different design than desired |
| 1. Addressing cancer prevention and control in Armenia: tobacco control and mHealth as key strategies. Int J Equity Health. 2021 | Different outcome than desired |
| 1. People living with type 1 diabetes point of view in COVID-19 times (COVIDT1 study): Disease impact, health system pitfalls and lessons for the future. Diabetes Res Clin Pract. 2021 | Different outcome than desired |
| 1. Can a telemonitoring system lead to decreased hospitalization in elderly patients? J Telemed Telecare. 2021 | Different outcome than desired |
| 1. Online interprofessional education related to chronic illness for health professionals: a scoping review. J Interprof Care. 2021 | Different design than desired |
| 1. Remote Application and Use of Real-Time Continuous Glucose Monitoring by Adults with Type 2 Diabetes in a Virtual Diabetes Clinic. Diabetes Technol Ther. 2021 | Different outcome than desired |
| 1. eHealth Technologies for Screening, Diagnosis, and Management of Viral Hepatitis: A Systematic Review. Clin Gastroenterol Hepatol. 2021 | Different design than desired |
| 1. Readiness and Acceptance of eHealth Services for Diabetes Care in the General Population: Cross-sectional Study. J Med Internet Res. 2021 | Different outcome than desired |
| 1. Human Wounds and Its Burden: An Updated Compendium of Estimates. Adv Wound Care (New Rochelle). 2019 | Different outcome than desired |
| 1. Telemonitoring type 1 diabetes patients during the COVID-19 pandemic in Brazil: was it useful? Arch Endocrinol Metab. 2021 | Different outcome than desired |
| 1. Description of e-Health Initiatives to Reduce Chronic Non-Communicable Disease Burden on Brazilian Health System. Int J Environ Res Public Health. 2021 | Different outcome than desired |
| 1. Smartphone-Based mHealth and Internet of Things for Diabetes Control and Self-Management. J Healthc Eng. 2021 | Different outcome than desired |
| 1. Telemedicine and urban diabetes during COVID-19 pandemic in Milano, Italy during lock-down: epidemiological and sociodemographic picture. Acta Diabetol. 2021 | Different outcome than desired |
| 1. mHealth as a primary mode of intervention for women at risk of, or diagnosed with, gestational diabetes: a scoping review protocol. JBI Evidence Synthesis. [2021](https://journals.lww.com/jbisrir/toc/2021/03000) 2. The Role of Telemedicine in Extending and Enhancing Medical Management of the Patient with Chronic Obstructive Pulmonary Disease. Medicina (Kaunas). 2021 | Different outcome than desired |
| 1. Use and preferences regarding internet-based health care delivery in patients with chronic kidney disease. BMC Med Inform Decis Mak. 2021 | Different population than desired |
| 1. Evaluation of a telemedicine program managing high-risk pregnant women with pre-existing diabetes in Arkansas's Medicaid program. Semin Perinatol. 2021 | Different outcome than desired |
| 1. Predictive performance and impact of algorithms in remote monitoring of chronic conditions: a systematic review and meta-analysis. International Journal of Medical Informatics. 2021 | Different design than desired |
| 1. Diabetes Management Delivery and Pregnancy Outcomes in Women with Gestational Diabetes Mellitus during the First Wave of the 2020 COVID-19 Pandemic: A Single-Reference Center Report. J Diabetes Res. 2021 | Different design than desired |
| 1. Integrated Measurement for Early Detection (MIDO) as a digital strategy for timely assessment of non-communicable disease profiles and factors associated with unawareness and control: a retrospective observational study in primary healthcare facilities in Mexico. BMJ Open. 2021 | Different outcome than desired |
| 1. Digital health technology for Indigenous older adults: A scoping review. International Journal of Medical Informatics. 2021 | Different outcome than desired |
| 1. The Rapid Transition to Telemedicine and Its Effect on Access to Care for Patients with Type 1 Diabetes During the COVID-19 Pandemic. Diabetes Care. 2021 | Different outcome than desired |
| 1. Use and perception of telemedicine in people with type 1 diabetes during the COVID-19 pandemic-Results of a global survey. Endocrinol Diabetes Metab. 2020 | Different outcome than desired |
| 1. Attitudes Toward Using COVID-19 mHealth Tools Among Adults with Chronic Health Conditions: Secondary Data Analysis of the COVID-19 Impact Survey. JMIR Mhealth Uhealth. 2020 | Different outcome than desired |
| 1. Association between the nurse-led program with mental health status, quality of life, and heart failure rehospitalization in chronic heart failure patients. Medicine (Baltimore). 2021 | Different outcome than desired |
| 1. Disparities in Telemedicine Use for Subspecialty Diabetes Care During COVID-19 Shelter-In-Place Orders. J Diabetes Sci Technol. 2021 | Different outcome than desired |
| 1. A model for national assessment of barriers for implementing digital technology interventions to improve hypertension management in the public health care system in India. BMC Health Serv Res. 2021 | Different outcome than desired |
| 1. Diabetes in COVID-19 pandemic-prevalence, patient characteristics and adverse outcomes. Int J Clin Pract. 2021 | Different outcome than desired |
| 1. Implementation of Digital Monitoring Services During the COVID-19 Pandemic for Patients with Chronic Diseases: Design Science Approach. J Med Internet Res. 2021 | Different outcome than desired |
| 1. Effectiveness of a pathway-driven eHealth-based integrated care model (PEICM) for community-based hypertension management in China: study protocol for a randomized controlled trial. Trials. 2021 | Different outcome than desired |
| 1. Managing Type 1 Diabetes among Saudi adults on insulin pump therapy during the COVID-19 lockdown. Diabetes Metab Syndr. 2021 | Different outcome than desired |
| 1. Telemedicine interventions for hypertension management in low- and middle-income countries: A scoping review. PLOS ONE. 2021 | Different design than desired |
| 1. Cross Sectional E-Health Evaluation Study for Telemedicine and M-Health Approaches in Monitoring COVID-19 Patients with Chronic Obstructive Pulmonary Disease (COPD). Int. J. Environ. Res. Public Health. 2021 | Different population than desired |
| 1. Internet Use, Electronic Health Literacy, and Hypertension Control among the Elderly at an Urban Primary Care Center in Thailand: A Cross-Sectional Study. Int J Environ Res Public Health. 2021 | Different outcome than desired |
| 1. Relationships Between Mobile eHealth Literacy, Diabetes Self-care, and Glycemic Outcomes in Taiwanese Patients with Type 2 Diabetes: Cross-sectional Study. JMRI. 2021 | Different outcome than desired |
| 1. Improvement in glycaemic control in paediatric and young adult type 1 diabetes patients during COVID-19 pandemic: role of telemedicine and lifestyle changes. Acta Biomed. 2021 | Different outcome than desired |
| 1. Preferences for mHealth Technology and Text Messaging Communication in Patients with Type 2 Diabetes: Qualitative Interview Study. J Med Internet Res. 2021 | Different outcome than desired |
| 1. Personalized hypertension management based on serial assessment and telemedicine (PHMA): a cluster randomize controlled trial protocol in Anhui, China. *BMC* Cardiovasc Disord. 2021 | Different outcome than desired |
| 1. The imperious need for telemedicine for the care of diabetes during the COVID-19 pandemic. A comprehensive approach study. Gaceta médica de Mexico. 2021 | Different outcome than desired |
| 1. Telemedicine satisfaction of primary care patients during COVID-19 pandemics]. Semergen. 2021 | Different outcome than desired |
| 1. Targeting of uncontrolled hypertension in the emergency department (TOUCHED): Design of a randomized controlled trial. Contemporary Clinical Trials. 2021 | Different outcome than desired |
| 1. Optimizing a digital intervention for managing blood pressure in stroke patients using a diverse sample: Integrating the person-based approach and patient and public involvement. Health Expectations. 2020 | Different outcome than desired |
| 1. Enhancing clinical judgement in virtual care for complex chronic disease. Journal of Clinical evaluation in Clinical practice. 2022 | Different outcome than desired |
| 1. A 15-month experience with a primary care-based telemedicine screening program for diabetic retinopathy. *BMC* Ophthalmol. 2021 | Different outcome than desired |
| 1. Diabetes self-management in online health communities: an information exchange perspective. BMC Med Inform Decis Mak. 2021 | Different outcome than desired |
| 1. The Telehealth Paradox in the Neediest Patients. J Natl Med Assoc. 2021 | Different outcome than desired |
| 1. An mHealth-Based Intervention for Adolescents with Type 1 Diabetes and Their Parents: Pilot Feasibility and Efficacy Single-Arm Study. JMIR Mhealth Uhealth. 2021 | Different outcome than desired |
| 1. A mobile health application to support self-management in patients with chronic obstructive pulmonary disease: a randomised controlled trial. *Clinical Rehabilitation*. 2021 | Different population than desired |
| 1. Content Analysis: First-Time Patient User Challenges with Top-Rated Commercial Diabetes Apps. Telemedicine and e-Health. 2021 | Different outcome than desired |
| 1. Telemedicine Use and Health-Related Concerns of Patients with Chronic Conditions During COVID-19: Survey of Members of Online Health Communities. J Med Internet Res. 2021 | Different outcome than desired |
| 1. Effectiveness of a Mobile Health and Self-Management App for High-Risk Patients with Chronic Obstructive Pulmonary Disease in Daily Clinical Practice: Mixed Methods Evaluation Study. JMIR Mhealth Uhealth. 2021 | Different outcome than desired |
| 1. Follow-up with Telemedicine in Early Discharge for COPD Exacerbations: Randomized Clinical Trial (TELEMEDCOPD-Trial). COPD. 2021 | Different population than desired |
| 1. Remote-Management of COPD: Evaluating the Implementation of Digital Innovation to Enable Routine Care (RECEIVER): the protocol for a feasibility and service adoption observational cohort study. BMJ Open Respir Res. 2021 | Different population than desired |
| 1. Systematically developing a family-based health promotion intervention for women with prior gestational diabetes based on evidence, theory and co-production: the Face-it study. BMC Public Health. 2021 | Different outcome than desired |
| 1. Effects of eHealth-Based Multiple Health Behavior Change Interventions on Physical Activity, Healthy Diet, and Weight in People with Noncommunicable Diseases: Systematic Review and Meta-analysis. J Med Internet Res. 2021 | Different outcome than desired |
| 1. A Prospective, Non-randomized Feasibility and Preliminary Efficacy Study of a Telemedicine-Enabled Co-management Intervention for Adults With Type 2 Diabetes and Moderate Anxiety and/or Depression. *The Science of Diabetes Self-Management and Care*. 2021 | Different outcome than desired |
| 1. Changes to care delivery at nine international pediatric diabetes clinics in response to the COVID-19 global pandemic. Pediatr Diabetes. 2021 | Different outcome than desired |
| 1. Effects of nurse-led web-based interventions on people with type 2 diabetes mellitus: A systematic review and meta-analysis. J Telemed Telecare. 2021 | Different outcome than desired |
| 1. The impact of a prolonged lockdown and use of telemedicine on glycemic control in people with type 1 diabetes during the COVID-19 outbreak in Saudi Arabia. Diabetes Res Clin Pract. 2021 | Different outcome than desired |
| 1. Same same-but different: using qualitative studies to inform concept elicitation for quality-of-life assessment in telemedical care: a request for an extended working model. Health Qual Life Outcomes. 2021 | Different outcome than desired |
| 1. A Web-Based Computer-Tailored Program to Improve Treatment Adherence in Patients with Type 2 Diabetes: Randomized Controlled Trial. J Med Internet Res. 2021 | Different outcome than desired |
| 1. Impact of telemedicine on health outcomes in children with medical complexity: an integrative review. Eur J Pediatr. 2021 | Different design than desired |
| 1. The Effect of Noninvasive Telemonitoring for Chronic Heart Failure on Health Care Utilization: Systematic Review. J Med Internet Res. 2021 | Different design than desired |
| 1. Tecnologias digitais no cuidado a pessoas com diabetes durante a pandemia de COVID-19: revisão de escopo. | Different design than desired |
| 1. The value of physical examination in the era of telemedicine. J R Coll Physicians Edinb. 2021 | Different outcome than desired |
| 1. Effect of a Digital Intervention on Depressive Symptoms in Patients with Comorbid Hypertension or Diabetes in Brazil and Peru: Two Randomized Clinical Trials. JAMA. 2021 | Different outcome than desired |
| 1. Perceptions of Using Multiple Mobile Health Devices to Support Self-Management Among Adults with Type 2 Diabetes: A Qualitative Descriptive Study. J Nurs Scholarsh. 2021 | Different population than desired |
| 1. Telemedicine for Children With Medical Complexity: A Randomized Clinical Trial. Pediatrics. 2021 | Different population than desired |
| 1. Type 2 Diabetes Prevention Focused on Normalization of Glycemia: A Two-Year Pilot Study. Nutrients. 2021 | Different outcome than desired |
| 1. Telemedicine in diabetic retinopathy screening in India. Indian J Ophthalmol. 2021 | Different outcome than desired |
| 1. Impact of COVID-19 lockdown on flash and real-time glucose sensor users with type 1 diabetes in England. Acta Diabetol. 2021 | Different outcome than desired |
| 1. Comparing the Efficacies of Telemedicine and Standard Prenatal Care on Blood Glucose Control in Women with Gestational Diabetes Mellitus: Randomized Controlled TrialJMIR Mhealth Uhealth 2021 | Different outcome than desired |
| 1. Effects of the Physician-Primary-Healthcare Nurse Telemedicine Model (P-NTM) on Medication Adherence and Health-Related Quality of Life (HRQoL) of Patients with Chronic Disease at Remote Rural Areas. Int J Environ Res Public Health. | Different outcome than desired |
| 1. Specialist to non-specialist teleconsultations in chronic respiratory disease management: A systematic review. | Different population than desired |
| 1. Pulmonary Rehabilitation in a Post-COVID-19 World: Telerehabilitation as a New Standard in Patients with COPD. Int J Chron Obstruct Pulmon Dis. 2021 | Different population than desired |
| 1. Effect of Telemedicine Dietary Intervention for Endothelial Function in Patients with Type 2 Diabetes Mellitus on Mediterranean Diet. Isr Med Assoc J. 2021 | Different outcome than desired |
| 1. Effects of Telemedicine and mHealth on Systolic Blood Pressure Management in Stroke Patients: Systematic Review and Meta-Analysis of Randomized Controlled Trials. JMIR Mhealth Uhealth. 2021 | Different design than desired |
| 1. Following the COVID-19 Experience, Many Patients with Type 1 Diabetes Wish to Use Telemedicine in a Hybrid Format. Int J Environ Res Public Health. 2021 | Different design than desired |
| 1. Diabetic Foot Disease during the COVID-19 Pandemic. Medicina (Kaunas). 2021 | Different outcome than desired |
| 1. Efficiency of an mHealth App and Chest-Wearable Remote Exercise Monitoring Intervention in Patients With Type 2 Diabetes: A Prospective, Multicenter Randomized Controlled Trial. JMIR Mhealth Uhealth. 2021 | Different outcome than desired |
| 1. Telemedicine Chronic Viral Hepatitis C Treatment during the Lockdown Period in Romania: A Pilot Study. Int J Environ Res Public Health. 2021 | Different outcome than desired |
| 1. The use of mobile health interventions for gestational diabetes mellitus: a descriptive literature review. J Med Life. 2021 | Different outcome than desired |
| 1. Digital messaging to support control for type 2 diabetes (StAR2D): a multicentre randomised controlled trial. BMC Public Health. 2021 | Different outcome than desired |
| 1. Comparative effect of eHealth interventions on hypertension management-related outcomes: A network meta-analysis. Int J Nurs Stud. 2021 | Different design than desired |
| 1. Using telehealth for rural paediatric diabetics: Does it deliver good care? J Paediatr Child Health. 2021 | Different outcome than desired |
| 1. Good practices for dialysis education, treatment, and eHealth: A scoping review | Different design than desired |
| 1. Management of diabetes mellitus through teleconsultation during COVID-19 and similar scenarios - Guidelines from Indian Council of Medical Research (ICMR) expert group. Diabetes Metab Syndr. 2021 | Different outcome than desired |
| 1. Clinical Effectiveness of Different Technologies for Diabetes in Pregnancy: Systematic Literature Review J Med Internet Res 2021 | Different design than desired |
| 1. Mean and visit-to-visit variability of glycated hemoglobin, and the risk of non-alcoholic fatty liver disease. J Diabetes Investig. 2021 | Different design than desired |
| 1. BPM Support for Patient-Centred Clinical Pathways in Chronic Diseases. Sensors (Basel). 2021 | Different design than desired |
| 1. Effectiveness of eHealth Interventions in Improving Medication Adherence for Patients with Chronic Obstructive Pulmonary Disease or Asthma: Systematic Review. J Med Internet Res. 2021 | Different design than desired |
| 1. Pandemic-Associated Trends in Measurement of HbA1c in Children with Diabetes Mellitus and Validation of Dried Blood Spot as an Alternative Sample Matrix. Ann Clin Lab Sci. 2021 | Different outcome than desired |
| 1. 2021 Spanish Society of Hypertension position statement about telemedicine. Hipertens Riesgo Vasc. 2021 | Different outcome than desired |
| 1. Coronavirus Disease 2019 and Kidney Transplantation in Saudi Arabia: Outcomes and Future Opportunities. Ann Transplant. 2021 | Different outcome than desired |
| 1. mHealth: Where Is the Potential for Aiding Informal Caregivers? Stud Health Technol Inform. 2021 | Different outcome than desired |
| 1. Telemedicine HCV treatment in department of corrections results in high SVR in era of direct-acting antivirals. | Different outcome than desired |
| 1. A Teaching Kitchen Medical Groups Visit with an eHealth Platform for Hypertension and Cardiac Risk Factors: A Qualitative Feasibility Study. J Altern Complement Med. 2021 | Different outcome than desired |
| 1. Personalization of Conversational Agent-Patient Interaction Styles for Chronic Disease Management: Two Consecutive Cross-sectional Questionnaire Studies. J Med Internet Res. 2021 | Different outcome than desired |
| 1. Diabetes Care During COVID-19 Pandemic in Singapore Using a Telehealth Strategy. Horm Metab Res. 2021 | Different outcome than desired |
| 1. Effectiveness of a Nurse-Led Tele-Homecare Program for Patients with Multiple Chronic Illnesses and a High Risk for Readmission: A Randomized Controlled Trial. J Nurs Scholarsh. 2021 | Different outcome than desired |
| 1. Impact of pharmacist intervention for blood pressure control in patients with chronic kidney disease: A meta-analysis of randomized clinical trials. J Clin Pharm Ther. 2021 | Different outcome than desired |
| 1. Narrative Analysis of the Impact of COVID-19 on Patients with Chronic Obstructive Pulmonary Disease, Their Caregivers, and Healthcare Professionals in Italy. Int J Chron Obstruct Pulmon Dis. 2021 | Different outcome than desired |
| 1. Diabetes and Technology in the Covid-19 Pandemic Crisis. J Diabetes Sci Technol. 2021 | Different outcome than desired |
| 1. Tailored or adapted interventions for adults with chronic obstructive pulmonary disease and at least one other long-term condition: a mixed methods review. Cochrane Database Syst Rev. 2021 | Different design than desired |
| 1. SAFE@HOME: Cost analysis of a new care pathway including a digital health platform for women at increased risk of preeclampsia. Pregnancy Hypertens. 2021 | Different outcome than desired |
| 1. Mobile health application usage and quality of care at a hypertension clinic: an observational cohort study. J Hypertens. 2021 | Different outcome than desired |
| 1. The Effectiveness of Telemedical Monitoring Program DiabCare Tirol for Patients with Gestational Diabetes Mellitus. Stud Health Technol Inform. 2021 | Different outcome than desired |
| 1. Teleassistance for Patients with Type 1 Diabetes During the COVID-19 Pandemic: Results of a Pilot Study. J Med Internet Res. 2021 | Different outcome than desired |
| 1. Web-Based Self-management Program (SPACE for COPD) for Individuals Hospitalized with an Acute Exacerbation of Chronic Obstructive Pulmonary Disease: Nonrandomized Feasibility Trial of Acceptability. JMIR Mhealth Uhealth. 2021 | Different population than desired |
| 1. Complex interventions to implement a diabetic retinopathy care pathway in the public health system in Kerala: The Nayanamritham study protocol. BMJ Open. 2021 | Different outcome than desired |
| 1. The experience of Australian general practice patients at high risk of poor health outcomes with telehealth during the COVID-19 pandemic: a qualitative study. BMC Fam Pract. 2021 | Different outcome than desired |
| 1. Lessons Learnt during the COVID-19 Pandemic: For Patients with End-Stage Renal Disease, We Should Prioritize Home-Based Treatment and Telemedicine. Kidney Blood Press Res. 2021 | Different outcome than desired |
| 1. The Need for Ethnoracial Equity in Artificial Intelligence for Diabetes Management: Review and Recommendations. J Med Internet Res. 2021 | Different outcome than desired |
| 1. Measuring Success of Patients' Continuous Use of Mobile Health Services for Self-management of Chronic Conditions: Model Development and Validation. J Med Internet Res. 2021 | Different outcome than desired |
| 1. Are residents receiving the training needed within their residency programs to optimally manage patients with diabetes? Postgrad Med. 2021 | Different outcome than desired |
| 1. Evaluation of an mHealth-enabled hierarchical diabetes management intervention in primary care in China (ROADMAP): A cluster randomized trial. PLoS Med. 2021 | Different outcome than desired |
| 1. A qualitative study of clinician perceptions regarding the potential role for digital health interventions for the management of COPD. Health Informatics J. 2021 | Different population than desired |
| 1. "In my age, we didn't have the computers": Using a complexity lens to understand uptake of diabetes eHealth innovations into primary care-A qualitative study. PLoS One. 2021 | Different outcome than desired |
| 1. Is a cloud-based platform useful for diabetes management in Colombia? The Tidepool experience. Comput Methods Programs Biomed. 2021 | Different outcome than desired |
| 1. A multidisciplinary Telehealth approach for community dwelling older adults. Geriatr Nurs. 2021 | Different outcome than desired |
| 1. Assessing the Impact of Telemonitoring-Facilitated Lifestyle Modifications on Diabetes Outcomes: A Systematic Review and Meta-Analysis. Telemed J E Health. 2021 | Different design than desired |
| 1. Patient and supporter factors affecting engagement with diabetes telehealth. Am J Manag Care. 2021 | Different outcome than desired |
| 1. Comparison of hepatitis C treatment outcomes between telehepatology and specialty care clinics in the era of direct-acting antivirals. J Telemed Telecare. 2021 | Different population than desired |
| 1. Mobile Health-Based Thermometer for Monitoring Wound Healing After Endovascular Therapy in Patients with Chronic Foot Ulcer: Prospective Cohort Study. JMIR Mhealth Uhealth. 2021 | Different outcome than desired |
| 1. The Nourish Protocol: A digital health randomized controlled trial to promote the DASH eating pattern among adults with hypertension. Contemp Clin Trials. 2021 | Different outcome than desired |
| 1. Identifying critical features of type two diabetes prevention interventions: A Delphi study with key stakeholders. PLoS One. 2021 | Different outcome than desired |
| 1. Effect of mobile health based peripartum management of gestational diabetes mellitus on postpartum diabetes: A randomized controlled trial. Diabetes Res Clin Pract. 2021 | Different outcome than desired |
| 1. Supervised maintenance programmes following pulmonary rehabilitation compared to usual care for chronic obstructive pulmonary disease. Cochrane Database Syst Rev. 2021 | Different population than desired |
| 1. Contactless: a new personalised telehealth model in chronic pediatric diseases and disability during the COVID-19 era. Ital J Pediatr. 2021 | Different outcome than desired |
| 1. On the efficacy of behavior change techniques in mHealth for self-management of diabetes: A meta-analysis. J Biomed Inform. 2021 | Different design than desired |
| 1. Domiciliary Cough Monitoring for the Prediction of COPD Exacerbations. Lung. 2021 | Different population than desired |
| 1. An Implementation Framework for Telemedicine to Address Noncommunicable Diseases in Thailand. Asia Pac J Public Health. 2021 | Different outcome than desired |
| 1. A Longitudinal Perspective on User Uptake of an Electronic Personal Health Record for Diabetes, With Respect to Patient Demographics. J Diabetes Sci Technol. 2021 | Different outcome than desired |
| 1. Health-related quality of life associated with diabetic retinopathy in patients at a public primary care service in southern Brazil. Arch Endocrinol Metab. 2021 | Different outcome than desired |
| 1. Efficacy of eHealth Interventions for Adults with Diabetes: A Systematic Review and Meta-Analysis. Int J Environ Res Public Health. 2021 | Different design than desired |
| 1. nternet-Based Medication Management Services Improve Glycated Hemoglobin Levels in Patients with Type 2 Diabetes. Telemed J E Health. 2021 | Different outcome than desired |
| 1. Reduction of hypoglycaemia, lifestyle modifications and psychological distress during lockdown following SARS-CoV-2 outbreak in type 1 diabetes. Diabetes Metab Res Rev. 2021 | Different outcome than desired |
| 1. Home blood pressure monitoring in the diagnosis and treatment of hypertension: a systematic review. Am J Hypertens. 2011 | Different design than desired |
| 1. Telehealth-delivered CBT-I programme enhanced by acceptance and commitment therapy for insomnia and hypnotic dependence: A pilot randomized controlled trial. J Sleep Res. 2021 | Different outcome than desired |
| 1. Care of Pediatric Patients with Diabetes During the Coronavirus Disease 2019 (COVID-19) Pandemic. Pediatr Clin North Am. 2021 | Different outcome than desired |
| 1. Epidemiological characteristics and therapeutic management of patients with chronic heart failure who use smartphones: Potential impact of a dedicated smartphone application (report from the OFICSel study). Arch Cardiovasc Dis. 2021 | Different outcome than desired |
| 1. The Effectiveness of Virtual Training on the MiniMed™ 670G System in People with Type 1 Diabetes During the COVID-19 Pandemic. Diabetes Technol Ther. 2021 | Different outcome than desired |
| 1. Effectiveness of diabetes education including insulin injection technique and dose adjustment through telemedicine in hospitalized patients with COVID-19. Diabetes Metab Syndr. 2021 | Different outcome than desired |
| 1. Barriers in establishing systematic diabetic retinopathy screening through telemedicine in low- and middle-income countries. Indian J Ophthalmol. 2021 | Different outcome than desired |
| 1. Effects of hybrid comprehensive telerehabilitation on cardiopulmonary capacity in heart failure patients depending on diabetes mellitus: subanalysis of the TELEREH-HF randomized clinical trial | Different outcome than desired |
| 1. Sustaining Gains in Diabetic Eye Screening: Outcomes from a Stakeholder-Based Implementation Program for Teleophthalmology in Primary Care. Telemed J E Health. 2021 | Different outcome than desired |
| 1. Process evaluation of a brief messaging intervention to improve diabetes treatment adherence in sub-Saharan Africa. BMC Public Health. 2021 | Different outcome than desired |
| 1. Glycaemic control in the paediatric and young adult population with type 1 diabetes following a single telehealth visit - what have we learned from the COVID-19 lockdown? Acta Diabetol. 2021 | Different outcome than desired |
| 1. Key toolkits of non-pharmacological management in COPD: during and beyond COVID-19. Front Biosci (Landmark Ed). 2021 | Different design than desired |
| 1. Sex differences in risk factors for depressive symptoms in patients with COPD: The 2014 and 2016 Korea National Health and Nutrition Examination Survey. BMC Pulm Med. 2021 | Different population than desired |
| 1. Blood pressure monitoring should be a habit": adaptation of the Check. Change. Control. program for Asian American older adults, from group-based in-person to one-on-one telephone delivery. Transl Behav Med. 2021 | Different outcome than desired |
| 1. Challenges for Non-COVID Patients with Chronic Kidney Disease in Bangladesh: An Observation during Coronavirus Disease Pandemic. *INQUIRY:* The Journal of Health Care Organization, Provision, and Financing. 2021 | Different population than desired |
| 1. Tele-Monitoring System for Chronic Diseases Management: Requirements and Architecture. Int J Environ Res Public Health. 2021 | Different outcome than desired |
| 1. Nocturnal Hypoglycaemia in Patients with Diabetes Mellitus: Database Analysis of a Cohort Using Telemedicine Support for Self-Monitoring of Blood Glucose over a 10-Year-Long Period. Medicina (Kaunas). 2021 | Different outcome than desired |
| 1. COVID-19 Pandemic and Virtual Clinics for Diabetes Care. Diabetes Technol Ther. 2021 | Different outcome than desired |
| 1. Mobile Health Salt Reduction Intervention for People with Hypertension: Results of a Feasibility Randomized Controlled Trial. JMIR Mhealth Uhealth. 2021 | Different outcome than desired |
| 1. Designing internet-enabled patient education for self-management of T2D diabetes-The case of the Razavi-Khorasan province in Iran. PLoS One. 2021 | Different outcome than desired |
| 1. Prescribing of SGLT2 inhibitors in primary care: A qualitative study of General Practitioners and Endocrinologists. Diabetes Res Clin Pract. 2021 | Different outcome than desired |
| 1. La télémédecine, d'ETAPES à COVIDOM …vers une nouvelle ère ? [Telemedicine from experimentation (ETAPES) to COVIDOM… a new era ?]. Ann Cardiol Angeiol (Paris). 2021 | Different outcome than desired |
| 1. Telehealth strategy to mitigate the negative psychological impact of the COVID-19 pandemic on type 2 diabetes: A randomized controlled trial. Acta Diabetol. 2021 | Different outcome than desired |
| 1. COVID-19-Related Downscaling of In-Hospital Liver Care Decreased Patient Satisfaction and Increased Liver-Related Mortality | Different outcome than desired |
| 1. Understanding factors critical to the implementation of ehealth in chronic disease management: a realist review protocol. BMJ Open. 2021 | Different outcome than desired |
| 1. Management of diabetes in elderly patients during the COVID-19 pandemic: current and future perspectives. Expert Rev Endocrinol Metab. 2021 | Different outcome than desired |
| 1. A Novel User Utility Score for Diabetes Management Using Tailored Mobile Coaching: Secondary Analysis of a Randomized Controlled Trial. JMIR Mhealth Uhealth. 2021 | Different outcome than desired |
| 1. Real-world insights from launching remote peer-to-peer mentoring in a safety net healthcare delivery setting, Journal of the American Medical Informatics Association. 2021 | Different outcome than desired |
| 1. The Performance of Digital Monitoring Devices for Oxygen Saturation and Respiratory Rate in COPD: A Systematic Review. COPD. 2021 | Different population than desired |
| 1. Diabetes Care and Glycemic Control During the COVID-19 Pandemic in the United States. JAMA Intern Med. 2021 | Different outcome than desired |
| 1. A Comprehensive 6A Framework for Improving Patient Self-Management of Hypertension Using mHealth Services: Qualitative Thematic Analysis. J Med Internet Res. 2021 | Different outcome than desired |
| 1. Telehealth mitigates COPD disease progression compared to standard of care: a randomized controlled crossover trial. J Intern Med. 2021 | Different population than desired |
| 1. Why #WeAreNotWaiting-Motivations and Self-Reported Outcomes Among Users of Open-source Automated Insulin Delivery Systems: Multinational Survey. J Med Internet Res. 2021 | Different outcome than desired |
| 1. Feasibility of an online platform delivery of pulmonary rehabilitation for individuals with chronic respiratory disease. BMJ Open Respir Res. 2021 | Different outcome than desired |
| 1. The Sinonasal Outcome Test-22 or European Position Paper: Which Is More Indicative of Imaging Results? Otolaryngol Head Neck Surg. 2021 | Different outcome than desired |
| 1. Application of 5G Technology to Conduct Real-Time Teleretinal Laser Photocoagulation for the Treatment of Diabetic Retinopathy. JAMA Ophthalmol. 2021 | Different outcome than desired |
| 1. I fear COVID but diabetic foot (DF) is worse: a survey on patients' perception of a telemedicine service for DF during lockdown. Acta Diabetol. 2021 | Different outcome than desired |
| 1. Impact of home telemonitoring and management support on blood pressure control in non-dialysis CKD: a systematic review protocol. BMJ Open. 2021 | Different design than desired |
| 1. Adherence to Telemonitoring Therapy for Medicaid Patients with Hypertension: Case Study. J Med Internet Res. 2021 | Different design than desired |
| 1. Identifying molecular insight of synergistic complexities for SARS-CoV-2 infection with pre-existing type 2 diabetes. Comput Biol Med. 2021 | Different outcome than desired |
| 1. Through the Storm: Automated Peritoneal Dialysis with Remote Patient Monitoring during COVID-19 Pandemic. Blood Purif. 2021 | Different outcome than desired |
| 1. Telemedicine in children with medical complexity on home ventilation during the COVID-19 pandemic. Pediatr Pulmonol. 2021 | Different outcome than desired |
| 1. Follow-Up Compliance for Patients Diagnosed with Diabetic Retinopathy After Teleretinal Imaging in Primary Care. Telemed J E Health. 2021 | Different outcome than desired |
| 1. Retaining diverse adults with diabetes in a long-term trial: Strategies, successes, and lessons learned. Contemp Clin Trials. 2021 | Different outcome than desired |
| 1. Virtual triage and outcomes of diabetic foot complications during Covid-19 pandemic: A retro-prospective, observational cohort study. PLoS One. 2021 | Different outcome than desired |
| 1. Phone-Based Intervention under Nurse Guidance after Stroke (PINGS II) Study: Protocol for a Phase III Randomized Clinical Trial. J Stroke Cerebrovasc Dis. 2021 | Different population than desired |
| 1. Virtual training on the hybrid close loop system in people with type 1 diabetes (T1D) during the COVID-19 pandemic. Diabetes Metab Syndr. 2021 | Different outcome than desired |
| 1. Program to Avoid Cerebrovascular Events through Systematic Electronic Tracking and Tailoring of an Eminent Risk factor: Protocol of a RCT. Journal of Stroke and Cerebrovascular Diseases. 2021 | Different population than desired |
| 1. Perception of virtual clinics among Saudi adults with type 1 diabetes during the COVID-19 pandemic. Diabetes Metab Syndr. 2021 | Different outcome than desired |
| 1. Diabetes Study Group of the Italian Society for Pediatric Endocrinology and Diabetes. The effect of the COVID-19 pandemic on telemedicine in pediatric diabetes centers in Italy: Results from a longitudinal survey. Diabetes Res Clin Pract. 2021 | Different outcome than desired |
| 1. Implementation of Teleretinal Screening Using Optical Coherence Tomography in the Veterans Health Administration. Telemed J E Health. 2021 | Different population than desired |
| 1. A new interventional home care model for COVID management: Virtual Covid IP. Diabetes Metab Syndr. 2021 | Different population than desired |
| 1. Prognosis Score System to Predict Survival for COVID-19 Cases: a Korean Nationwide Cohort Study J Med Internet Res. 2021 | Different population than desired |
| 1. Evaluating the Potential Use of Smartphone Apps for Diabetes Self-Management in an Underserved Population: A Qualitative Approach. Int J Environ Res Public Health. 2021 | Different outcome than desired |
| 1. Analysis of Gender Differences in HRV of Patients with Myalgic Encephalomyelitis/Chronic Fatigue Syndrome Using Mobile-Health Technology. Sensors (Basel). 2021 | Different population than desired |
| 1. Patient Adherence to Hemoglobin A1c Testing Recommendations in Telemedicine and In-Office Cohorts During COVID-19. JAMA Netw Open. 2021 | Different outcome than desired |
| 1. Adapting to telemedicine in the COVID-19 era: Feasibility of dried blood spot testing for hemoglobin A1c. Diabetes Metab Syndr. 2021 | Different outcome than desired |
| 1. Evaluation Methods Used to Assess mHealth Applications for Cardiovascular Disease: First Results of a Scoping Review. Stud Health Technol Inform. 2021 | Different population than desired |
| 1. Exploring Real-World mHealth Use for Diabetes Consultations: Pros and Pitfalls of a Pragmatic Mixed-Methods Approach. Stud Health Technol Inform. 2021 | Different outcome than desired |
| 1. Personalized Type 2 Diabetes Management Using a Mobile Application Integrated with Electronic Medical Records: An Ongoing Randomized Controlled Trial. Int J Environ Res Public Health. 2021 | Different outcome than desired |
| 1. Adherence to an eHealth Self-Management Intervention for Patients with Both COPD and Heart Failure: Results of a Pilot Study. Int J Chron Obstruct Pulmon Dis. 2021 | Different population than desired |
| 1. Artificial Intelligence Can Improve Patient Management at the Time of a Pandemic: The Role of Voice Technology. J Med Internet Res. 2021 | Different outcome than desired |
| 1. Principles for virtual health care to deliver real equity in diabetes. The Lancet Diabetes & Endocrinology. 2021 | Different outcome than desired |
| 1. A Virtual Training Program for the Tandem t:slim X2 Insulin Pump: Implementation and Outcomes. Diabetes Technol Ther. 2021 | Different outcome than desired |
| 1. Assessment of Glycosylated Hemoglobin Outcomes Following an Enhanced Medication Therapy Management Service via Telehealth. Int J Environ Res Public Health. 2021 | Different outcome than desired |
| 1. The Association of Mobile Health Applications with Self-Management Behaviors among Adults with Chronic Conditions in the United States. Int J Environ Res Public Health. 2021 | Different outcome than desired |
| 1. Determinants of penetrance and variable expressivity in monogenic metabolic conditions across 77,184 exomes. Nat Commun. 2021 | Different population than desired |
| 1. Discordant health beliefs and telehealth use in African Americans with diabetes. J Am Geriatr Soc. 2021 | Different outcome than desired |
| 1. Diabetes Self-Management Education Support Healthy Teaching Kitchen Cooking Classes Delivered Using a Telehealth Technology. The Science of Diabetes Self-Management and Care. 2021 | Different outcome than desired |
| 1. Covid-19: A critical time for cross-sector social work care management. Soc Work Health Care. 2021 | Different outcome than desired |
| 1. A two-layer probabilistic model to predict COPD exacerbations for patients in telehealth. Comput Biol Med. 2021 | Different population than desired |
| 1. Effect of Layperson-Delivered, Empathy-Focused Program of Telephone Calls on Loneliness, Depression, and Anxiety Among Adults During the COVID-19 Pandemic: A Randomized Clinical Trial. Jama Psyquiatry. 2021 | Different outcome than desired |
| 1. The impact of the involvement of a healthcare professional on the usage of an eHealth platform: a retrospective observational COPD study. Respir Res. 2021 | Different population than desired |
| 1. Sequential Multiple Assignment Randomized Trial (SMART) to identify optimal sequences of telemedicine interventions for improving initiation of insulin therapy: A simulation study. BMC Med Res Methodol. 2021 | Different outcome than desired |
| 1. Innovations to Sustain Non-Communicable Disease Services in the Context of COVID-19: Report from Pakkred District, Nonthaburi Province, Thailand. Glob Heart. 2021 | Different outcome than desired |
| 1. Improving Efficiency of the Barbershop Model of Hypertension Care for Black Men with Virtual Visits. J Am Heart Assoc. 2021 | Different outcome than desired |
| 1. Facilitating engagement of persons with opioid use disorder in treatment for hepatitis C virus infection via telemedicine: Stories of onsite case managers. J Subst Abuse Treat. 2021 | Different population than desired |
| 1. Cerebral Tissue Oxygenation Saturation Does Not Equate with Cerebral Oxygen Availability. Respiration. 2021 | Different population than desired |
| 1. Thoracic Pain and Pericardial Effusion in a Patient With Chronic Pancreatitis. Gastroenterology. 2021 | Different population than desired |
| 1. Diabetic retinopathy screening - Widen the net, tighten the mesh. Indian J Ophthalmol. 2021 | Different outcome than desired |
| 1. The impact of a telemedicine intervention on home non-invasive ventilation in a rural population with advanced COPD. Clin Respir J. 2021 | Different population than desired |
| 1. Development of a Smartphone-Based Optical Device to Measure Hemoglobin Concentration Changes for Remote Monitoring of Wounds. Biosensors (Basel). 2021 | Different outcome than desired |
| 1. Virtual interprofessional chronic cough clinic: An efficient and appealing approach to a complex problem. Int Forum Allergy Rhinol. 2021 | Different population than desired |
| 1. Impact of telephone support programme using telemonitoring on stage of change towards healthy eating and active exercise in people with prediabetes. J Telemed Telecare. 2021 | Different outcome than desired |
| 1. Follow-Up of a Massive Open Online Course in Type 2 Diabetes Self-Management Education. J Diabetes Sci Technol. 2021 | Different outcome than desired |
| 1. Review of nomograms to counsel patients after oncologic surgery: a support for telemedicine to stratify the risk of relapse and customize the follow-up scheduling. Minerva Urol Nephrol. 2021 | Different population than desired |
| 1. Is it safe to start steroids at home for a COPD exacerbation after virtual assessment in the COVID-19 era? Cleve Clin J Med. 2021 | Different population than desired |
| 1. Trends in Pain Medication Initiation Among Patients with Newly Diagnosed Diabetic Peripheral Neuropathy, 2014-2018. JAMA Netw Open. 2021 | Different outcome than desired |
| 1. Evaluating the effectiveness and utility of a novel culturally adapted telemonitoring system in improving the glycaemic control of Asians with type-2 diabetes mellitus: a mixed method study protocol. Trials. 2021 | Different outcome than desired |
| 1. Assessing the Therapeutic Utility of Professional Continuous Glucose Monitoring in Type 2 Diabetes Across Various Therapies: A Retrospective Evaluation. Adv Ther. 2017 | Different outcome than desired and different period |
| 1. Mobile phone-based remote patient monitoring system for management of hypertension in diabetic patients. Am J Hypertens. 2007 | Different outcome than desired and different period |
| 1. Are there time and cost savings by using telemanagement for patients on intensified insulin therapy? A randomised, controlled trial. Comput Methods Programs Biomed. 2002 | Different outcome than desired and different period |
| 1. Telecare of diabetic patients with intensified insulin therapy. A randomized clinical trial. Stud Health Technol Inform. 2000 | Different outcome than desired and different period |
| 1. Evaluating the effectiveness and utility of a novel culturally adapted telemonitoring system in improving the glycaemic control of Asians with type-2 diabetes mellitus: a mixed method study protocol. Trials. 2021 | Different outcome than desired |
| 1. Disease-Management-Programs in the Field of Diabetes Mellitus with Identification of the Best Practice in Europe: A Scoping Review. Horm Metab Res. 2020 | Different outcome than desired |
| 1. Mobile Apps for Health Behavior Change: Protocol for a Systematic Review. JMIR Res Protoc. 2020 | Different design than desired |
| 1. The Patient-centered Medical Home as an Intervention Strategy for Diabetes Mellitus: A Systematic Review of the Literature. Curr Diabetes Rev. 2021 | Different design than desired |
| 1. An implementation framework and a feasibility evaluation of a clinical decision support system for diabetes management in secondary mental healthcare using CogStack. BMC Med Inform Decis Mak. 2022 | Different outcome than desired |
